# Hydrogen sulfide acts as a sulfur source for iron sulfur cluster biosynthesis in cysteine desulfurase-deficient *Escherichia coli* under anaerobic conditions

**DOI:** 10.3389/fmicb.2026.1759970

**Published:** 2026-03-11

**Authors:** Heng Li, Jun Wang, Xiaorui Li, Guanya Jia, Haisheng Gan, Yanxiong Wang, Zhiwei Ma, Zhilong Zhu, Xiaoya Shang, Weining Niu

**Affiliations:** School of Life Sciences, Northwestern Polytechnical University, Xi’an, Shaanxi, China

**Keywords:** anaerobic conditions, cysteine desulfurase IscS, *Escherichia coli*, hydrogen sulfide, iron–sulfur cluster, scaffold protein IscU

## Abstract

The cysteine desulfurase (IscS) is a core component of the ISC iron–sulfur (Fe-S) cluster assembly system in *Escherichia coli*. Deficiency of IscS leads to serious growth defects in *E. coli*, along with reduced activity of Fe-S cluster-dependent enzymes. We previously demonstrated that the growth defect of IscS-deficient *E. coli* (Δ*iscS*) is completely restored by H₂S exposure, but the underlying molecular mechanism was not fully understood. Here, based on proteomic analysis, we identified 19 up-regulated Fe-S proteins in the Δ*iscS* mutant upon H₂S exposure, 13 of which are involved with energy metabolism. Correspondingly, H₂S exposure also enhanced the activity of Fe-S enzymes in the mutant. Metabolomic analysis revealed a remarkable increase in the levels of the energy metabolites NAD^+^, succinate, and leucine. These results implied that H_2_S could restore cell proliferation and Fe-S cluster biosynthesis by compensating for the functional loss of IscS. We also constructed a series of mutants, each lacking a single component of the ISC assembly system. A key observation was that the Δ*iscU* mutant, deficient in the Fe-S cluster scaffold protein IscU, failed to have its growth defect rescued by H₂S exposure. These findings indicated that H_2_S promotes Fe-S cluster biosynthesis on IscU, ruling out direct assembly on apoproteins. Moreover, Na₂S supplementation during recombinant expression of aconitase B in the Δ*iscS* mutant significantly increased its Fe-S cluster abundance and enzymatic activity. We also demonstrated that, unlike the Δ*iscS* mutant, deletion of *sufS*, which encodes the cysteine desulfurase of the SUF Fe-S cluster biogenesis system, did not significantly impair bacterial growth, and the resulting mutant’s proliferation was not affected by H₂S exposure. Our study elucidates the mechanism by which H₂S exposure rescues the proliferation impairment of the *ΔiscS* mutant. Specifically, we demonstrate that H₂S functions as a sulfur donor for Fe-S cluster assembly, thereby compensating for the biosynthetic deficit.

## 1 Introduction

Iron–sulfur (Fe-S) clusters are among the oldest biological cofactors in nature (Srour et al., 2020; Shi et al., 2021). They are usually coordinated to polypeptides through cysteine residues and serve as redox centers, non-redox catalytic centers, or environmental sensors (Esquilin-Lebron et al., 2021). In cells, Fe-S cluster containing proteins play crucial roles in many important biological functions, such as electron transport, regulation of gene expression, and DNA repair (Srour et al., 2020; Esquilin-Lebron et al., 2021; Shi et al., 2021). Fe-S clusters can spontaneously assemble *in vitro* from iron and sulfide ions under anaerobic conditions, but *in vivo* assembly involves complex and highly regulated systems (Freibert et al., 2018). In *Escherichia coli* (*E. coli*), there are two main systems for Fe-S cluster biosynthesis, namely the ISC and SUF systems. Previous studies demonstrated that the ISC system is a house-keeping system, while the SUF system generally functions under stress conditions such as oxidative stress and iron limitation (Takahashi and Tokumoto, 2002; Bandyopadhyay et al., 2008; Tanaka et al., 2016; Tanaka et al., 2019). Deletion of the *iscS* gene encoding the cysteine desulfurase (IscS), which is the key protein responsible for transsulfuration in the ISC system (Smith et al., 2001; Urbina et al., 2001), not only results in a reduction in the activity of iron–sulfur cluster-dependent enzymes but also leads to serious growth defects of *E. coli* mutants (Schwartz et al., 2000; Outten et al., 2004; Tanaka et al., 2016; Wang et al., 2019).

In *E. coli*, the ISC system primarily comprises cysteine desulfurase (IscS), scaffold protein (IscU), A-type protein (IscA), iron donor (IscX), DnaJ-like co-chaperone (HscB), DnaK-like chaperone protein HscA, and ferredoxin (Fdx) (Py and Barras, 2010). IscS is a pyridoxal 5′-phosphate (PLP)-dependent enzyme that catalyzes the conversion of cysteine to alanine and sulfane sulfur *via* the formation of a protein-bound cysteine persulfide intermediate on a conserved cysteine residue and subsequently delivers sulfur to the scaffold protein IscU (Baussier et al., 2020; Braymer et al., 2021; Fujishiro et al., 2022). During sulfur transfer from IscS to IscU, Fdx provides electrons to reduce the persulfide intermediate to sulfide for Fe-S cluster biosynthesis (Gervason et al., 2025a). However, to date, the source of the iron in the biosynthesis of Fe-S clusters remains controversial. Some reports suggested that IscX protein transports iron to the scaffold protein IscU (Kim et al., 2014), while others proposed that IscA may act as an iron donor (Johnson et al., 2005; Esquilin-Lebron et al., 2021). Finally, the assembled Fe-S clusters on the scaffold protein IscU are rapidly transferred to the target apoproteins with the assistance of HscB and HscA (Vickery and Cupp-Vickery, 2007; Puglisi and Pastore, 2018). In mammals, the core components of Fe-S cluster biosynthesis include the cysteine desulfurase NFS1 (the ortholog of *E. coli* IscS), frataxin (FXN), the accessory protein ISD11, the scaffold protein ISCU, and the acyl carrier protein (ACP) (Zhang et al., 2021). Mutations in the genes encoding the sulfur transfer-related proteins NFS1, FXN, and ISD11 are associated with the human diseases, including infantile mitochondrial complex II/III deficiency, Friedreich’s ataxia (FRDA), and combined oxidative phosphorylation defects, respectively (Zhang et al., 2021).

Our previous studies demonstrated that the Δ*iscS* mutant cells exhibited significantly reduced H_2_S production and severe growth defects under anaerobic conditions compared to wild-type *E. coli* (Wang et al., 2019). Interestingly, the growth defect was completely restored in the presence of Na_2_S (an H_2_S donor), but not by the addition of cysteine, sodium sulfite, or sodium sulfate (Wang et al., 2019). However, the underlying molecular mechanism remains unclear. A key function of IscS is the provision of sulfur using cysteine as a substrate in Fe-S cluster biogenesis, and deletion of the *iscS* gene appears to affect sulfur supply (Mihara and Esaki, 2002; Das et al., 2021). We speculated that exogenous H₂S may serve as a sulfur donor to promote Fe-S cluster biosynthesis, thereby enhancing the activity of iron–sulfur cluster-dependent enzymes and restoring cell proliferation in the Δ*iscS* mutant. In fact, previous studies have established that sulfide can serve as a sulfur source for Fe-S cluster assembly. This includes both chemical reconstitution of apo-proteins (Kennedy and Beinert, 1988; Freibert et al., 2018) and *in vivo* assembly within methanogenic archaea, which live in high-sulfide environments and naturally lack cysteine desulfurase (Liu et al., 2010; Dussouchaud et al., 2025). Based on these literature reports, and reinforced by observations in our lab, we hypothesized that exogenous H_2_S could function as a sulfur donor for Fe-S cluster assembly in the Δ*iscS* mutant cells.

In this study, we further explored the mechanism by which exogenous H_2_S rescues the growth defect of the Δ*iscS* mutant under anaerobic conditions. By combining proteomic analysis, metabolomic analysis, Fe-S enzyme activity assays, and gene deletion analysis, we demonstrated that H_2_S can serve as a sulfur donor to promote Fe-S cluster biosynthesis in the Δ*iscS* mutant, thus enhancing cellular respiration and energy metabolism, and consequently restoring the growth defect of the Δ*iscS* mutant cells.

## 2 Materials and methods

### 2.1 Bacterial strains, plasmids, and chemicals

Strains and plasmids were listed in Supplementary Table S1. pEcCas (Addgene plasmid # 73227) and pEcgRNA (Addgene plasmid # 166581) were kindly provided by Sheng Yang from the Chinese Academy of Science (Li et al., 2021). *E. coli* strains were cultivated either in Luria-Bertani (LB) broth or in M9 minimal medium supplemented with 0.4% (w/v) glucose, thiamine, and trace elements (Cai et al., 2019). Unless otherwise specified, LB broth was used as the culture medium throughout this study. Antibiotics were used at the following concentrations: kanamycin (50 μg/mL), spectinomycin (50 μg/mL), and ampicillin (50 μg/mL). Unless otherwise specified, the chemicals were purchased from Sangon (Shanghai, China).

### 2.2 Proteomic analysis of the Δ*iscS* mutant with or without supplementation with 500 μM Na_2_S

Briefly, 5 mL of overnight culture was inoculated into a culture bottle containing 500 mL of LB medium with the appropriate chemicals as described in the text or figure legends. For anaerobic conditions, all the cultures were deoxygenated by nitrogen bubbling for 30 min, and *E. coli* was cultured in an anaerobic incubator at 37 °C with shaking (100 rpm). After a 3 h culture, the cells were harvested by centrifugation at 10,000 rpm for 10 min, and the supernatant was removed. Then cells were resuspended in normal saline (pH 7.0, 0.9% NaCl), followed by centrifugation to collect the precipitate as samples. Samples were then frozen in liquid nitrogen and set aside for later use.

Cell pellets were resuspended with RIPA Lysis and Extraction Buffer (Thermo Fisher Scientific, Waltham, MA, United States) and then homogenized completely with tissue grinders on ice. The protein concentration of each sample was determined using a BCA protein assay kit (TransGen Biotech, Beijing, China). After quantifying with the BCA protein kit, the protein sample (100 μg) was first diluted to 100 μL with 50 mM ammonium bicarbonate (NH₄HCO₃). Tris (2-carboxyethyl) phosphine (TCEP) was added to the protein samples at a final concentration of 10 mM, followed by incubation for 1 h at 55 °C. Next, the protein samples were alkylated with 20 mM iodoacetamide in the dark for 30 min. The protein was then precipitated by adding six volumes of pre-chilled acetone and freezing at −20 °C overnight. The protein precipitate was collected by centrifugation at 12,000 rpm for 10 min at 4 °C, and then redissolved in 100 μL of 50 mM NH₄HCO₃ solution. 2.5 μg of trypsin was added for overnight digestion at 37 °C. Subsequently, the tryptic peptides were desalted using a self-priming desalting column, and the solvent was evaporated in a vacuum centrifuge at 45 °C.

Dried peptides were dissolved in 0.1% formic acid and 2% acetonitrile in water and analyzed by liquid chromatography–tandem mass spectrometry (LC–MS/MS) using an Ultimate 3,000 RSLCnano system coupled to an Orbitrap Fusion Lumos Tribrid mass spectrometer (Thermo Fisher Scientific, Waltham, MA, United States). Samples were injected onto an Acclaim PepMap C18 column (75 μm i.d. × 150 mm, 3 μm particle size, 100 Å) (Thermo Fisher Scientific, Waltham, MA, United States) and separated over a 120-min method. The mobile phase was composed of solvent A (0.1% formic acid in 2% acetonitrile) and solvent B (0.1% formic acid in 80% acetonitrile) with gradient elution (0–5 min, 4% B; 5–85 min, 4–22% B; 85–110 min, 22–40% B; 110–111 min, 40–95% B; 111–120 min, 95% B) at a 300 nL/min flow rate. Survey full scan MS spectra (from m/z 300–1,400) were acquired with a resolution of 70,000. The peptides were analyzed with a 3e6 AGC target with a maximum injection time of 40 ms. The top 20 signal intensity ions were fragmented using high-energy collisional dissociation. MS/MS scans were obtained at a resolution of 17,500. The AGC target was set to 1e5, with a maximum injection time of 60 ms.

The raw MS files were analyzed and searched against an *E. coli* K12 database using MaxQuant software version 1.6.2.10 (Cox and Mann, 2008). The searched parameters were set as follows: carbamidomethyl (C) (fixed); oxidation (M) (variable); the enzyme was set to trypsin; maximum missed cleavages, 2; peptide mass tolerance, 20 ppm; fragment mass tolerance, 0.6 Da; and significance threshold, 0.05. The functional information of the proteins was analyzed in the UniProt database^1^. For the identification of differentially expressed proteins, the fold change of proteins ≥ 2 was considered upregulated, whereas the fold change of proteins ≤ 1/2 was considered downregulated. For Gene Ontology (GO) analysis, DAVID Bioinformatics Resources^2^ was used for GO term enrichment analysis to find significantly enriched biological processes (Huang et al., 2007). Pathway mapping of differentially expressed proteins was conducted using the Kyoto Encyclopedia of Genes and Genomes (KEGG) database^3^.

### 2.3 Metabolomic analysis of the Δ*iscS* mutant with or without supplementation with 500 μM Na_2_S

The cell samples cultured in the presence or absence of 500 μM Na_2_S were obtained as described in the proteomics analysis. Cell pellets were resuspended in 1 mL of pre-cooled methanol (−20 °C), and then sonicated for 1 h at 4 °C. After centrifugation at 12,000 rpm for 10 min, 800 μL of the supernatant was collected and vacuum-concentrated to evaporate the solvent. Subsequently, the extracted metabolites were redissolved in 100 μL of methanol, followed by centrifugation at 12,000 rpm for 10 min. A quality control (QC) sample was prepared by mixing aliquots from all supernatant samples.

Cellular metabolites were analyzed using a Nexera UHPLC LC-30A ultra-high-performance liquid chromatography (UHPLC) system (Shimadzu, Japan) coupled with a TripleTOF 5,600 + mass spectrometer (AB SCIEX, United States). The metabolites were separated by Waters HSS T3 C18 column (3 mm × 150 mm, 1.8 μm) with mobile phase A (0.1% formic acid in water) and mobile phase B (0.1% formic acid in acetonitrile) with a gradient elution (0–1 min, 5% B; 1–30 min, 5–95% B; 30–33 min, 95% B; 33–35 min, 95–5% B). The flow rate of the mobile phase was 0.2 mL/min, and the column temperature was maintained at 35 °C.

LC-ESI-MS/MS experiments were run in both positive and negative ion electrospray ionization modes. The mass range was set at m/z 100–1,500 using data-independent acquisition (DIA) mode. The parameters were set as follows: the capillary voltage was set to 5,000 V in the positive mode and 4,500 V in negative mode; capillary temperature was 500 °C; declustering potential was 60 V; collision energy (CE) was set to 35 V; and collision energy spread (CES) was 15 V. The data sets were processed using MS-DIAL software according to a study from Tsugawa et al. (2015). Multivariate data analyses such as principal component analysis (PCA) and partial least squares discriminant analysis (PLS-DA) were performed to determine the distributions and to find the metabolic differences between the control group and the experimental group. Differentially abundant metabolites were identified using variable importance for the projection (VIP) values (VIP > 1) and *t*-test (*p* < 0.05). Analytes were identified by database searches in the MassBank, ReSpect, and GNPS (global natural products social molecular networking) databases.

### 2.4 Construction of mutant strains

Unless otherwise specified, the mutant strains of *E. coli* BW25113 in this study were constructed using the method described by Li et al. (2021). The primers in this study are listed in Supplementary Table S2. The mutant *E. coli* strains were confirmed by sequencing PCR-amplified fragments from the flanking regions of each target site.

### 2.5 Measurement of growth curves

The overnight culture of *E. coli* was transferred to fresh LB medium with 1% (v/v) inoculum. For aerobic culture, *E. coli* was grown at 37 °C with shaking at 250 rpm. For anaerobic culture, the medium was deoxygenated by nitrogen bubbling for 30 min, and then *E. coli* was grown statically in sealed bottles at 37 °C. Cell growth was monitored spectrophotometrically by measurement of the optical density at 600 nm (OD_600_). The effect of Na_2_S (an H_2_S donor) on the growth of *E. coli* was determined according to our previous study (Wang et al., 2019).

### 2.6 RNA extraction and qPCR analysis

*Escherichia coli* cells in the exponential phase were harvested by centrifugation (10,000 rpm) for 10 min at 4 °C. Total RNA was extracted from *E. coli* cells by using the RNAiso Plus kit (TaKaRa, Dalian, China), and the concentration of RNA was measured using the Qubit 4 fluorometer (Thermo Fisher Scientific). Total RNA was reverse-transcribed into cDNA using the Evo M-MLV RT Kit with gDNA Clean for qPCR II (Accurate Biology, Changsha, China). Quantitative real-time PCR (qPCR) was performed using the SYBR Green Premix Pro Taq HS qPCR Kit (Accurate Biotechnology) according to the manufacturer’s instructions. Relative expression levels were calculated using the 2^−ΔΔCT^ method, with 16S rRNA serving as an endogenous control (Livak and Schmittgen, 2001). The primer sequences used for qPCR are shown in Supplementary Table S2.

### 2.7 Enzyme activity assays

The activity of nitrate reductase (NR) was determined using an NR assay Kit (Solarbio, Beijing, China). Briefly, bacterial cultures in the exponential growth phase were collected by centrifugation at 12,000 rpm for 10 min at 4 °C and washed three times with ice-cold 50 mM Tris–HCl buffer (pH 8.0), which was deoxygenated by nitrogen bubbling for 30 min. Next, the cell pellets were resuspended in 50 mM Tris–HCl buffer (pH 8.0) supplemented with lysozyme (0.5 mg/mL) and lysed by six freeze–thaw cycles in liquid nitrogen. The supernatant was obtained by centrifugation at 12,000 rpm for 15 min, and the protein concentration was determined using a BCA protein assay kit (TransGen Biotech, Beijing, China). The activity of nitrate reductase (NR) was measured by a multifunctional microplate reader according to the manufacturer’s instructions. One unit of activity was defined as the amount of enzyme that oxidizes 1 μmol of NADH per hour at 37 °C.

The fumarate reductase (FR) activity was determined by measuring the rate of NADH oxidation (Kim et al., 2016). The preparation of samples is consistent with that for the NR activity assay as described above. The protein concentration was determined using a BCA protein assay kit (TransGen Biotech, Beijing, China). The reaction mixture containing 50 mM Tris–HCl buffer (pH 8.0), 5 mM fumarate, and 0.5 mM NADH was added to a 96-well plate, and the reaction was initiated by the addition of the cell lysate supernatant. Change in the absorbance of NADH was monitored at 340 nm for 20 min at 1 min intervals. The reaction solution without fumarate was used as a negative control. One unit of activity was defined as the amount of enzyme that oxidizes 1 μmol of NADH per hour at 37 °C.

### 2.8 Reconstitution of Fe-S clusters in purified aconitase B (AcnB)

The gene *acnB* of *E. coli* aconitase B (Gene ID: 944864) was amplified by PCR using primers AcnB-F and AcnB-R (Supplementary Table S2). The PCR products were cloned into the pET28a plasmid to generate the expression plasmid pET28a-AcnB, which was subsequently transformed into *E. coli* BL21 (DE3) strain. Protein expression and purification were performed according to our previous study with a few modifications (Wang et al., 2019). Recombinant *E. coli* cells were cultured in LB medium with 50 mg/L kanamycin until the optical density at 600 nm (OD_600_) reached 0.6–0.8. The expression of aconitase B (AcnB) was induced by adding isopropyl β-D-1-thiogalactopyranoside (IPTG) to a final concentration of 0.1 mM, and incubation was continued for an additional 12 h at 30 °C. The harvested cells were resuspended in a lysis buffer containing 50 mM phosphate-buffered saline (PBS, pH 7.4), 500 mg/L lysozyme, 500 mM NaCl, 30 mM imidazole, and 1 mM phenylmethylsulfonyl fluoride (PMSF). After incubation on ice for 1 h, the resuspended cells were sonicated with an Ultrasonic Cell Disruption System (Scientz, Ningbo, China). Subsequently, the supernatant was collected by centrifugation at 12,000 rpm for 15 min at 4 °C and then loaded onto a HisTrap FF column (GE Healthcare, United States) pre-equilibrated with buffer (50 mM PBS buffer, pH 7.4, 500 mM NaCl, 30 mM imidazole). The recombinant AcnB was eluted with 300 mM imidazole in 50 mM PBS (pH 7.4), and the fractions containing AcnB were desalted using a HiTrap desalting column (GE Healthcare, United States) pre-equilibrated with 50 mM PBS (pH 7.4). The fractions containing the purified proteins were pooled and stored at −80 °C. The protein concentration was determined using a BCA protein assay kit (TransGen Biotech).

The apoprotein apo-AcnB was prepared according to a previous study (20). The recombinant AcnB (10 mg/mL), EDTA disodium (Na_2_EDTA), and potassium ferricyanide were added to the reaction solution (0.1 M Hepes, pH 7.5) at a molar ratio of 1:50:20, followed by incubation for 5–10 min at 4 °C until the complete loss of color from the cluster. The solution was desalted using a HiTrap desalting column (GE Healthcare, USA) pre-equilibrated with 50 mM Tris–HCl, pH 8.0. The eluted apoprotein apo-AcnB was frozen in aliquots and stored at −80 °C. Next, the Fe-S cofactors of the apoprotein apo-AcnB were reconstructed by chemical means anaerobically inside a glove box according to previous studies (Jordan et al., 1999; Freibert et al., 2018). The apo-AcnB (4 mg/mL) was incubated in deoxygenated buffer (50 mM Tris–HCl, pH 8.0, 50 mM NaCl, 5% glycerol, a 4-fold molar excess of both Na_2_S and Fe(NH_4_)_2_(SO_4_)_2_, 5 mM DTT) for 4 h at 37 °C. Then, the reconstituted protein was desalted using a HiTrap desalting column (GE Healthcare) pre-equilibrated with deoxygenated buffer (50 mM Tris–HCl, pH 8.0). The absorbance characteristics of Fe-S proteins were analyzed by a UV–vis spectrophotometer (Freibert et al., 2018).

### 2.9 Expression and purification of the Fe-S protein aconitase B (AcnB) in wild-type *E. coli* and the Δ*iscS* mutant

The PCR-amplified *acnB* gene was cloned into the pSE380 plasmid, generating the expression construct pSE380-AcnB. A 6 × His tag was then introduced at the carboxyl terminus of *acnB* by PCR-mediated site-directed mutagenesis. The resulting recombinant plasmid was transformed into two *E. coli* host strains: (1) BW25113 (WT) and (2) BW25113 Δ*iscS*. For the Na₂S treatment group, Na₂S was added hourly to a final concentration of 100 μM. Recombinant *E. coli* cells were cultured in LB medium supplemented with 50 mg/L ampicillin until the optical density at 600 nm (OD₆₀₀) reached 0.6–0.8. Protein expression was induced by adding 0.1 mM IPTG, followed by incubation for an additional 9 h at 30 °C. Protein purification was performed as described above using buffers that had been deoxygenated by nitrogen bubbling for 30 min. UV–visible absorption spectra of AcnB (1 mg/mL) were recorded from 250 to 700 nm using a microplate spectrophotometer (BioTek Synergy H1). A 200 μL aliquot of purified protein was loaded into a 96-well plate, with the corresponding protein-free buffer serving as the blank.

### 2.10 Assays of aconitase B (AcnB) activity

AcnB activity was determined by monitoring the formation of cis-aconitate from isocitrate at 240 nm in 50 mM Tris–HCl buffer (pH 8.0) containing citrate (20 mM) at 37 °C (Jordan et al., 1999). The cis-aconitate concentration was calculated using an extinction coefficient of 3,600 M^−1^ cm^−1^ at 240 nm. One unit of AcnB activity was defined as the amount of enzyme catalyzing the formation of 1 μmol cis-aconitate per minute at 37 °C.

### 2.11 Determination of NAD^+^ content

The intracellular NAD^+^ content was detected using a Coenzyme I NAD(H) Content Assay Kit (Solarbio, Beijing, China). Briefly, *E. coli* cells (approximately 5×10^6^ cells) in the exponential growth phase were collected by centrifugation at 10,000 rpm for 10 min at 4 °C. The cell pellets were resuspended in 500 μL of acidic extraction solution, and the cells were sonicated with an Ultrasonic Cell Disruption System (Scientz, Ningbo, China). Next, the lysis solution was boiled for 5 min and then cooled in an ice bath. After centrifugation at 12,000 rpm for 10 min at 4 °C, the supernatant was neutralized with an equal volume of alkaline solution, followed by centrifugation at 12,000 rpm for 10 min. The intracellular NAD^+^ content was determined according to the manufacturer’s instructions, and values were normalized to protein concentration.

### 2.12 Expression of wild-type IscS (WT) and the IscS (C328A) mutant in *E. coli* BW25113 (Δ*iscS*)

The *E. coli iscS* gene (Gene ID: 947004) was PCR-amplified using primers IscS-F and IscS-R (Supplementary Table S2) and cloned into the pBAD43 plasmid, generating the construct pBAD43-*iscS*. The pBAD43-*iscS*(C328A) mutant plasmid was subsequently generated from this template *via* PCR-mediated site-directed mutagenesis with primers F-328 and R-328. The empty vector (pBAD43) was chemically transformed into both *E. coli* BW25113 (wild type) and *E. coli* BW25113 Δ*iscS* strains. The recombinant plasmids pBAD43-*iscS* and pBAD43-*iscS*(C328A) were transformed into the *E. coli* BW25113 Δ*iscS* mutant. For growth assays, overnight culture aerobically was diluted to an OD₆₀₀ of ~1.0, then inoculated at 1% (v/v) inoculum into fresh LB medium containing spectinomycin and 0.2% arabinose. Cultures were incubated anaerobically at 37 °C for 9 h, and cell density was determined by spectrophotometry at 600 nm (OD_600_).

### 2.13 Detection of sulfane sulfur species

Sulfane sulfur species (e.g., persulfides/polysulfides) in the supernatants of lysed *E. coli* cells were quantified using the fluorescent probe SSP4 (3′,6′-di(O-thiosalicyl) fluorescein; Dojindo, Japan) (Bibli et al., 2018; Li et al., 2019). Sample preparation was identical to that described above for the NR activity assay. Protein concentrations were determined using a BCA assay kit, and all samples were normalized to a final concentration of 5 mg/mL. Aliquots (200 μL) of the normalized samples were then transferred to a 96-well plate, and SSP4 was added to a final concentration of 10 μM. After incubation in the dark at room temperature for 20 min, fluorescence intensities were measured at 515 nm (excitation 482 nm) using a multifunctional microplate reader. The fluorescence values were expressed as the intensity per mg of protein.

### 2.14 Determination of iron content

*Escherichia coli* cells were cultured in LB medium under anaerobic conditions until the optical density at 600 nm (OD_600_) reached ~ 0.2 (exponential growth phase). Then, bacterial cultures were adjusted to the same optical density (OD_600_) of 0.15. Subsequently, 8 mL of bacterial culture was centrifuged at 12,000 rpm for 5 min, and the cell pellets were washed three times with normal saline, followed by centrifugation to collect the cells. Next, the cells were treated with concentrated nitric acid at 70 °C for 2 h. Once the cells were completely nitrated, the temperature was increased to 150 °C until the nitric acid had completely evaporated. Subsequently, the samples were diluted to 10 mL with a solution containing 1% HNO₃ and 0.1% KCl, and the iron content was measured using atomic absorption spectroscopy (AAS; Analytik Jena AG, Jena, Germany) (Gruber et al., 2020; Zhang et al., 2021). The iron content was calculated based on the standard curve.

### 2.15 Statistical analyses

Data are represented as the means ± SD. Unpaired Student’s t test and one-way analysis of variance were employed to determine the statistical significance using GraphPad Prism 7.0. *p* < 0.05 was considered statistically significant.

## 3 Results

### 3.1 Exogenous H_2_S increased the levels of Fe-S proteins in the Δ*iscS* mutant

**Figure 1:**
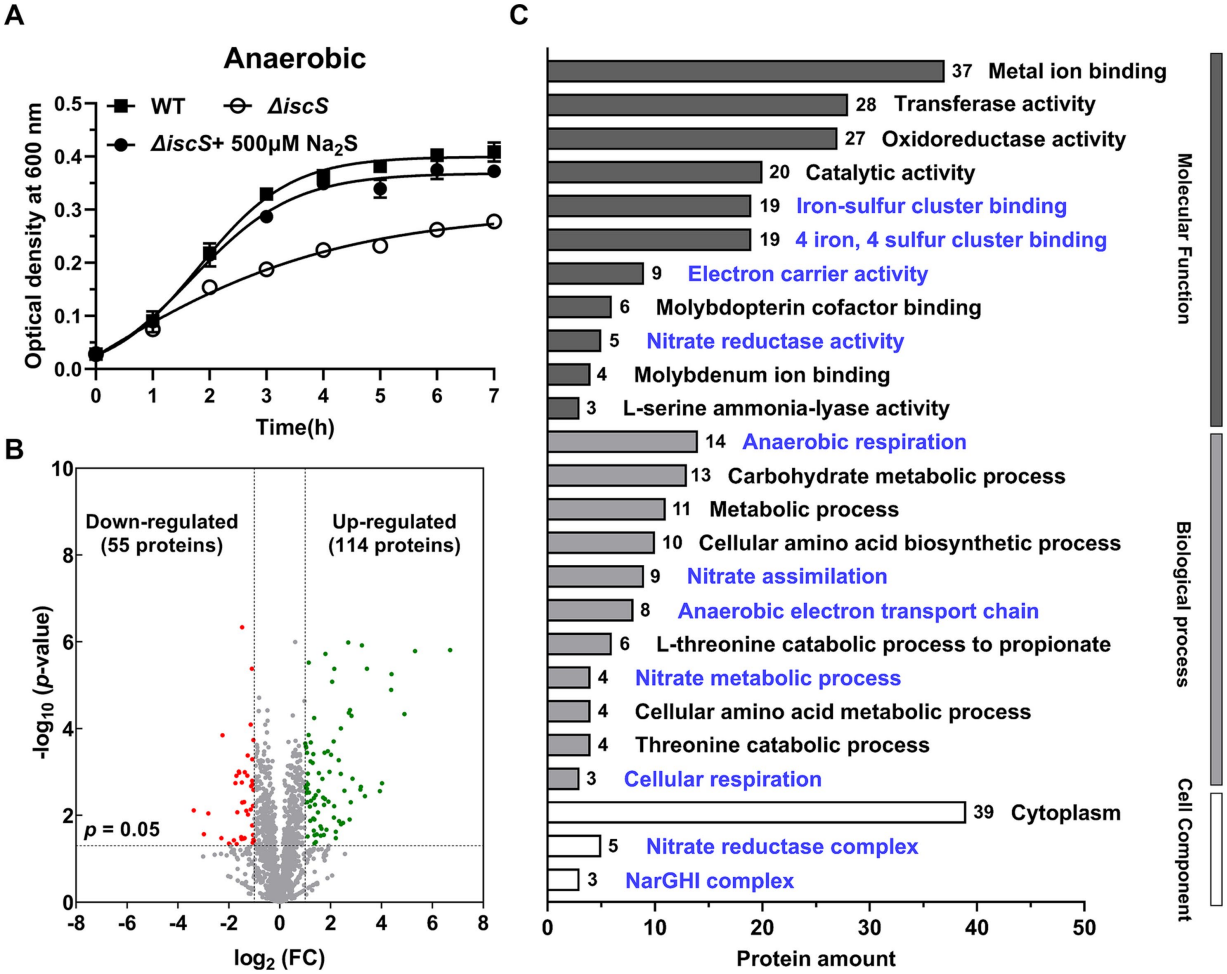
Exogenous H_2_S significantly altered the proteome of the Δ*iscS* mutant under anaerobic conditions. **(A)** The effect of 500 μM Na_2_S on the growth of the Δ*iscS* mutant cells grown in LB medium under anaerobic conditions. **(B)** Volcano plot depicting the analysis of the differentially expressed proteins in the Δ*iscS* mutant exposed to Na_2_S. Green and red dots represent significantly up-regulated and down-regulated proteins, respectively. Non-significant differences are represented by gray dots. **(C)** Gene ontology (GO) analysis of the up-regulated proteins. The blue font denotes the enriched proteins related to anaerobic respiration and the Fe-S cluster binding proteins.

Our previous study demonstrated that exogenous H_2_S promoted the growth and ATP synthesis of the Δ*iscS* mutant under anaerobic conditions (Wang et al., 2019). To reveal the molecular mechanism, we first performed proteomic analysis of the Δ*iscS* mutant cells cultured in the presence or absence of 500 μM Na_2_S (an H_2_S donor) (Figure 1A). A total of 2,171 proteins and 22,624 peptide fragments were identified. As shown in Supplementary Figure S1, the molecular weight distribution of proteins, peptide length distribution, the percentage of peptides with missed cleavage sites, HPLC separation of peptides, and mass spectrometry analysis indicated that the proteomic analysis was reliable. Exogenous H_2_S caused the up-regulation of 114 and down-regulation of 55 proteins in the ΔiscS mutant (Figure 1B).

Next, we analyzed the properties of the up-regulated proteins. As shown in Figure 1C, gene ontology (GO) analysis showed that most up-regulated proteins were related to the energy metabolism pathways under anaerobic conditions, especially nitrate respiration pathways. Since IscS primarily participates in the biosynthesis of Fe-S clusters, we focused our attention on the relevance of H_2_S to the levels of Fe-S proteins in the Δ*iscS* mutant. Interestingly, 19 Fe-S proteins were identified among the up-regulated proteins (Figure 1C), and their biological functions were presented in Table 1. These proteins are predominantly involved in anaerobic energy metabolism pathways, including nitrate and fumarate respiration, as well as electron transfer. In addition, the abundance of 3-isopropylmalate dehydratase large subunit and glutamate synthase [NADPH] small chain, which participate in leucine and glutamate biosynthesis, respectively, were increased (Table 1). Leucine and glutamate can be oxidized to α-ketoisocaproic acid and 2-oxoglutarate, respectively, and participate in energy metabolism in bacteria under anaerobic conditions (Díaz-Pérez et al., 2016; Buckel, 2021). Collectively, these results indicated that exogenous H_2_S increased the levels of Fe-S proteins, most of which are associated with anaerobic energy metabolism in the Δ*iscS* mutant.

**Table 1:** The up-regulated Fe-S proteins identified by proteomic analysis of the Δ*iscS* mutant strain exposed to H_2_S.

Protein names	Gene names	Metabolic pathway/function
Periplasmic nitrate reductase	*napA*	Nitrate respiration
Respiratory nitrate reductase 1 beta chain	*narH*	Nitrate respiration
Respiratory nitrate reductase 1 alpha chain	*narG*	Nitrate respiration
Nitrite reductase (NADH) large subunit	*nirB*	Nitrate respiration
Formate dehydrogenase, nitrate-inducible, major subunit	*fdnG*	Nitrate respiration
Formate dehydrogenase-O major subunit	*fdoG*	Nitrate respiration
Fumarate reductase iron–sulfur subunit	*frdB*	Fumarate respiration
Anaerobic glycerol-3-phosphate dehydrogenase subunit C	*glpC*	Anaerobic respiration
Hydrogenase-2 operon protein HybA	*hybA*	Anaerobic respiration
Dimethyl sulfoxide reductase DmsA	*dmsA*	Anaerobic respiration
NADH-quinone oxidoreductase subunit G	*nuoG*	Electron transfer
NADH-quinone oxidoreductase subunit I	*nuoI*	Electron transfer
Probable pyruvate-flavodoxin oxidoreductase	*ydbK*	Anaerobic glycolysis
3-isopropylmalate dehydratase large subunit	*leuC*	L-leucine biosynthesis
Glutamate synthase [NADPH] small chain	*gltD*	L-glutamate biosynthesis
L-serine dehydratase 2	*sdaB*	Amino acid metabolism
L-serine dehydratase TdcG	*tdcG*	Amino acid metabolism
NAD-dependent dihydropyrimidine dehydrogenase subunit PreT	*preT*	Nucleotide metabolism
Endonuclease III	*ntH*	DNA repair

### 3.2 Exogenous H₂S up-regulated genes encoding Fe-S proteins and enhanced their activity in the Δ*iscS* mutant

**Figure 2:**
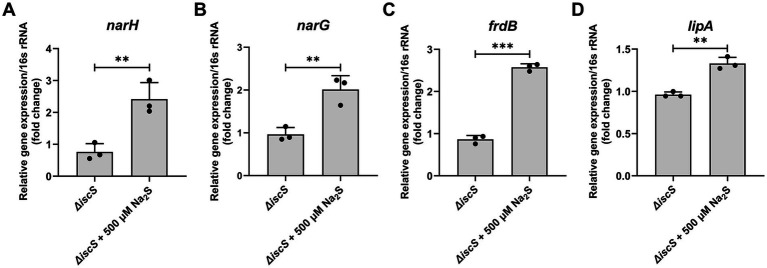
Effects of exogenous H₂S on the transcriptional levels of genes encoding Fe-S proteins in the Δ*iscS* mutant cells grown in LB medium under anaerobic conditions. **(A–D)** The effect of 500 μM Na_2_S on the transcription level of *narH*, *narG*, *frdB*, and *lipA*, respectively. Data are presented as means ± SD (*n* = 3). **p < 0.01, ***p < 0.001.

**Figure 3:**
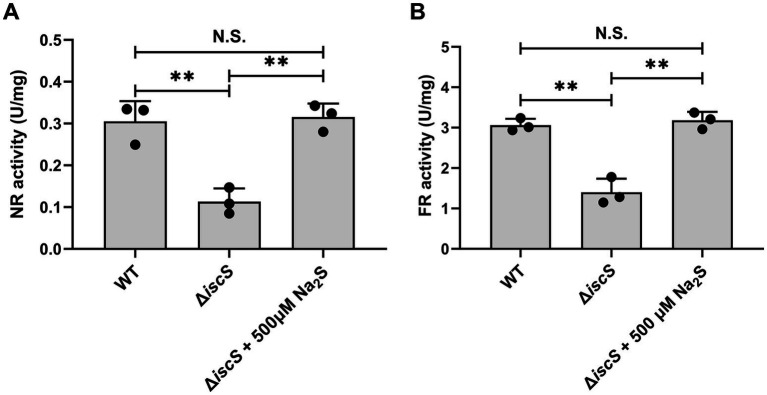
Effects of exogenous H_2_S on the activities of nitrate reductase (NR) **(A)** and fumarate reductase (FR) **(B)** in the Δ*iscS* mutant cells grown in LB medium under anaerobic conditions. Data are presented as means ± SD ((*n* = 3). **p < 0.01; N.S., not significant.

Based on the proteomic data, we selected several Fe-S proteins and measured the transcript levels of the corresponding genes and/or their enzyme activities. Consistent with the proteomic data, quantitative real-time polymerase chain reaction (qPCR) analysis showed that H₂S exposure significantly increased the transcript levels of *narH* (encoding respiratory nitrate reductase 1 beta chain) and *narG* (respiratory nitrate reductase 1 alpha chain) under anaerobic conditions (Figures 2A,B). Additionally, H₂S treatment significantly elevated the transcript levels of *frdB* (which encodes the fumarate reductase iron–sulfur subunit, playing a pivotal role as complex II in the electron transport chain) and *lipA* (involved in lipoic acid synthesis) (Figures 2C,D). Previous studies have established that the *E. coli* transcription factor YgaV responds to sulfide compounds to regulate anaerobic respiratory gene expression (Balasubramanian et al., 2022). Given this context, it is plausible that in the Δ*iscS* mutant, H₂S treatment may promote the expression of anaerobic metabolic pathway genes by modifying YgaV. We next assessed the effect of H_2_S exposure on the activities of two Fe-S enzymes in the Δ*iscS* mutant. As shown in Figure 3, the activities of nitrate reductase (NR) and fumarate reductase (FR) were completely restored to wild-type levels. These results suggested that exogenous H₂S enhanced the abundance and enzymatic activities of these Fe-S proteins in the Δ*iscS* mutant under anaerobic conditions.

### 3.3 Exogenous H₂S increased the metabolite levels of NAD^+^, leucine, and succinate in the Δ*iscS* mutant

**Figure 4:**
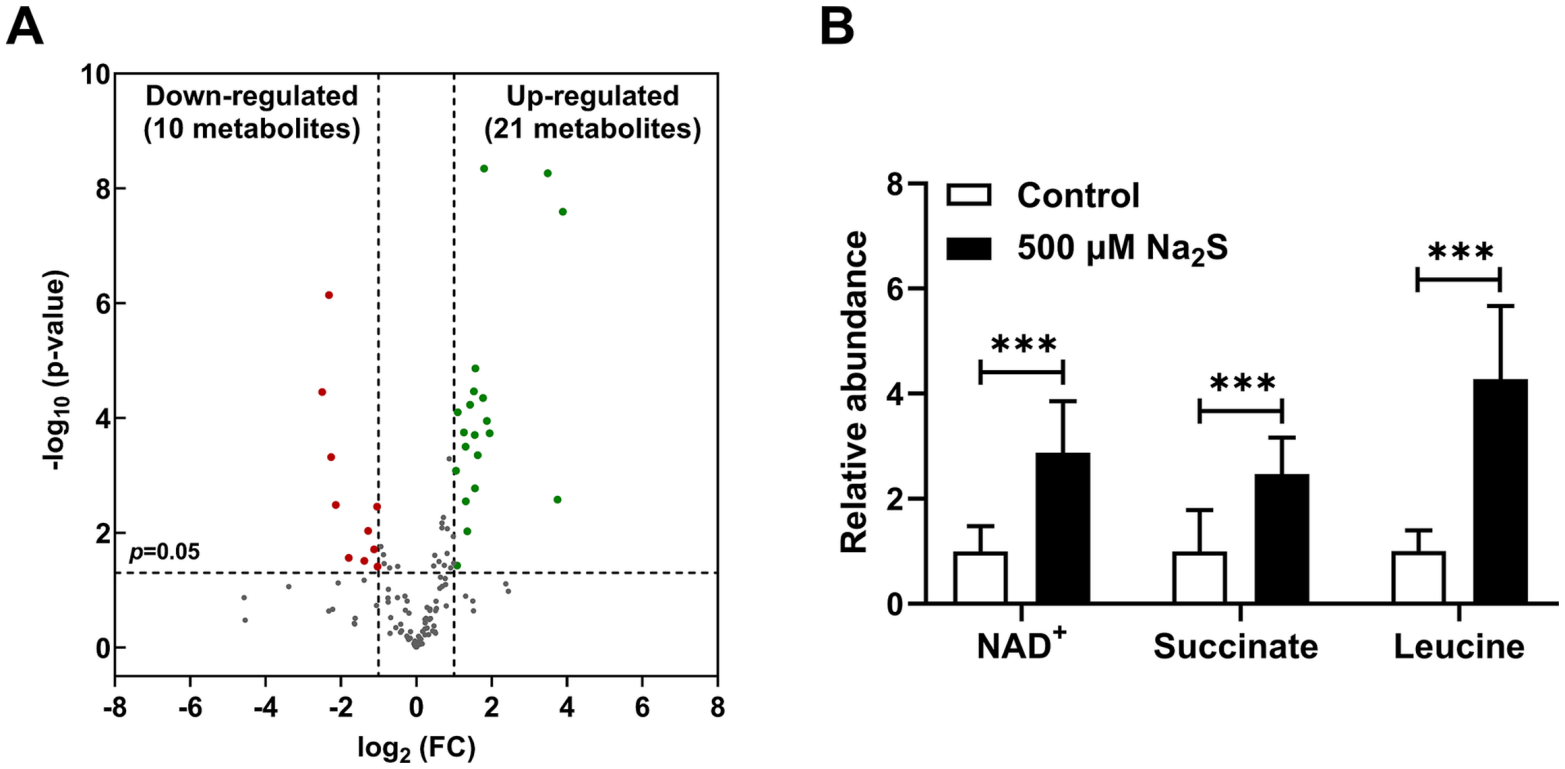
Exogenous H_2_S altered the metabolism of the Δ*iscS* mutant under anaerobic conditions. **(A)** Volcano plot showing differential metabolites between the Na_2_S-treated group and the control group in the Δ*iscS* mutant cells grown in LB medium under anaerobic conditions. Green and red dots represent significantly up-regulated and down-regulated metabolites, respectively. Nonsignificant differences are represented by gray dots. **(B)** Relative abundance of NAD^+^, succinate, and leucine in *E. coli* (Δ*iscS*) mutant cells exposed to Na_2_S compared to the control group (without Na_2_S treatment). Data are presented as means ± SD (*n* = 10). ***p < 0.001.

To further explore the molecular mechanism by which H_2_S exposure promoted energy metabolism and restored the growth defect in the Δ*iscS* mutant, we performed metabolomic analysis on the Δ*iscS* mutant cells cultured with or without 500 μM Na_2_S. Both principal component analysis (PCA) and partial least squares-discriminant analysis (PLS-DA) showed clear separation between the two groups, demonstrating the high reliability of the metabolomic data (Supplementary Figure S2). As shown in Figure 4A, 21 metabolites were significantly up-regulated and 10 were down-regulated. Interestingly, levels of NAD^+^, leucine, and succinate were significantly up-regulated compared with the control group (Figure 4B). The elevated content of NAD^+^, a key electron acceptor, suggests an accelerated metabolic rate in *E. coli* (Unden et al., 1994). In addition, the observed increase in leucine is functionally important, as it can be oxidized to α-ketoisocaproic acid and utilized in energy metabolism under anaerobic conditions (Díaz-Pérez et al., 2016). This increase is likely explained by our proteomic data (Table 1), which showed a significant increase in the abundance of the Fe-S protein 3-isopropylmalate dehydratase large subunit. In addition, measurement of NAD^+^ levels in the Δ*iscS* mutant revealed a significant increase upon H₂S treatment, confirming our metabolomic data (Supplementary Figure S3).

**Figure 5:**
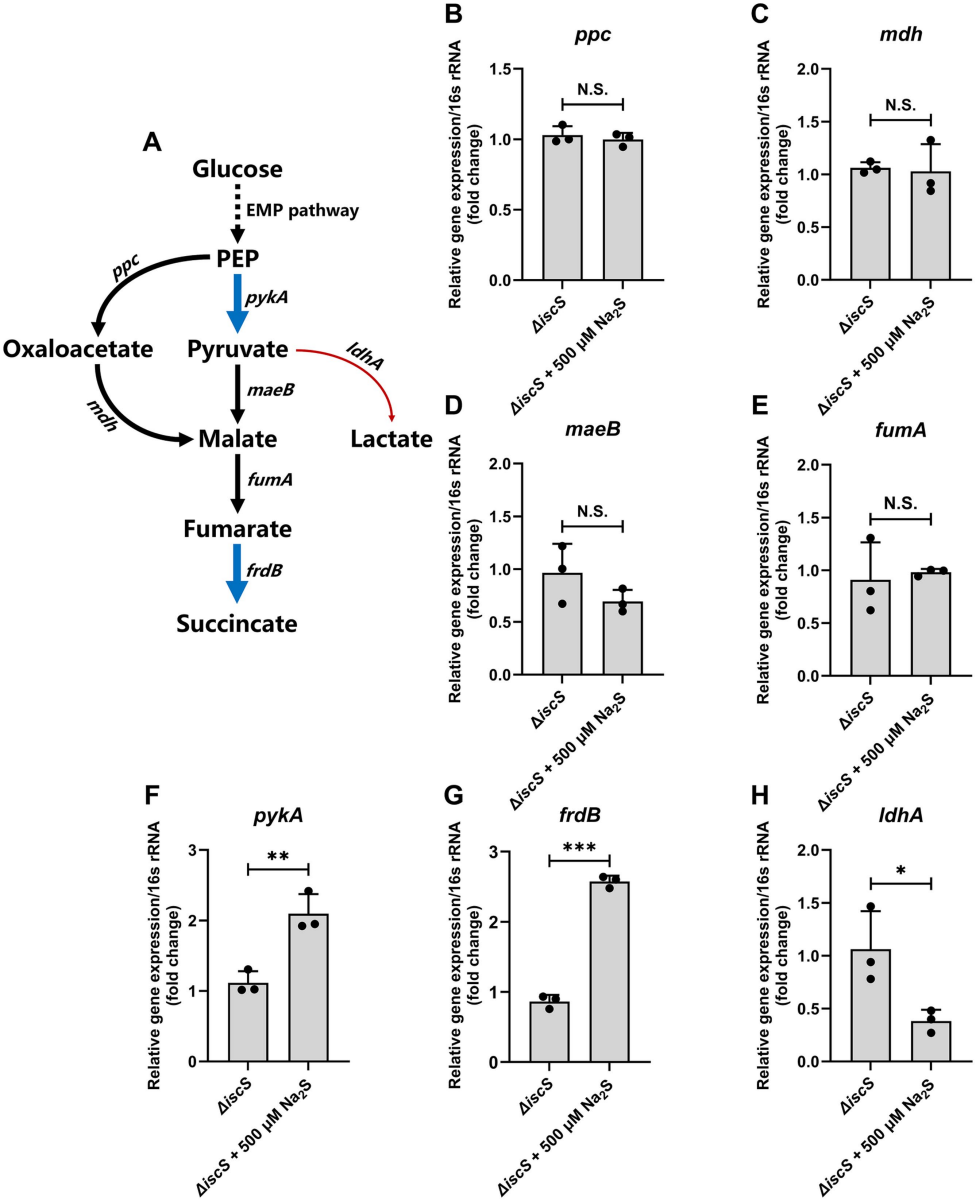
Effects of exogenous H_2_S on the transcription level of genes involved in succinate metabolism under anaerobic conditions. **(A)** Schematic diagram of the metabolic pathways for succinate production from glucose in *E. coli* under anaerobic conditions. Gene names are shown in italics, and blue and red arrows represent the genes whose transcription was significantly up-regulated or down-regulated, respectively, in the Δ*iscS* mutant cells exposed to 500 μM Na_2_S in LB medium under anaerobic conditions. Non-significant differences are represented by black arrow. **(B–H)** The effect of 500 μM Na_2_S on the transcription level of *ppc*, *mdH*, *maeB*, *fumA*, *pykA*, *frdB*, and *ldhA* in the Δ*iscS* mutant cells, respectively. Data are presented as means ± SD (*n* = 3). *p < 0.05, **p < 0.01, ***p < 0.001. N.S., not significant.

Given that succinate is the final product of fumarate respiration in *E. coli* under anaerobic conditions (Lee et al., 2005), and we observed its significant accumulation in H₂S-exposed Δ*iscS* cells, we examined the transcription levels of genes in the succinate metabolism pathway (Figure 5A) (Vemuri et al., 2002; Lee et al., 2005). H₂S exposure did not significantly alter the transcription of *ppc* (phosphoenolpyruvate carboxylase), *mdh* (malate dehydrogenase), *maeB* (NADP-dependent malic enzyme), or *fumA* (fumarate hydratase class I) (Figures 5B–E). However, it significantly up-regulated *pykA* (pyruvate kinase) and *frdB* (fumarate reductase iron–sulfur subunit) (Figures 5F,G), while down-regulating *ldhA* (D-lactate dehydrogenase), which is involved in lactate metabolism (Figure 5H). The increased abundance of pyruvate kinase—the enzyme catalyzing the rate-limiting, ATP-generating step of glycolysis—diverted more phosphoenolpyruvate to pyruvate rather than oxaloacetate under anaerobic conditions. The increased pyruvate flux was diverted from lactate fermentation to fumarate respiration, significantly elevating succinate content (Figure 5A), a finding consistent with our metabolomic analysis.

### 3.4 H_2_S can act as a sulfur donor to participate in the assembly of Fe-S clusters in the Δ*iscS* mutant cells

**Figure 6:**
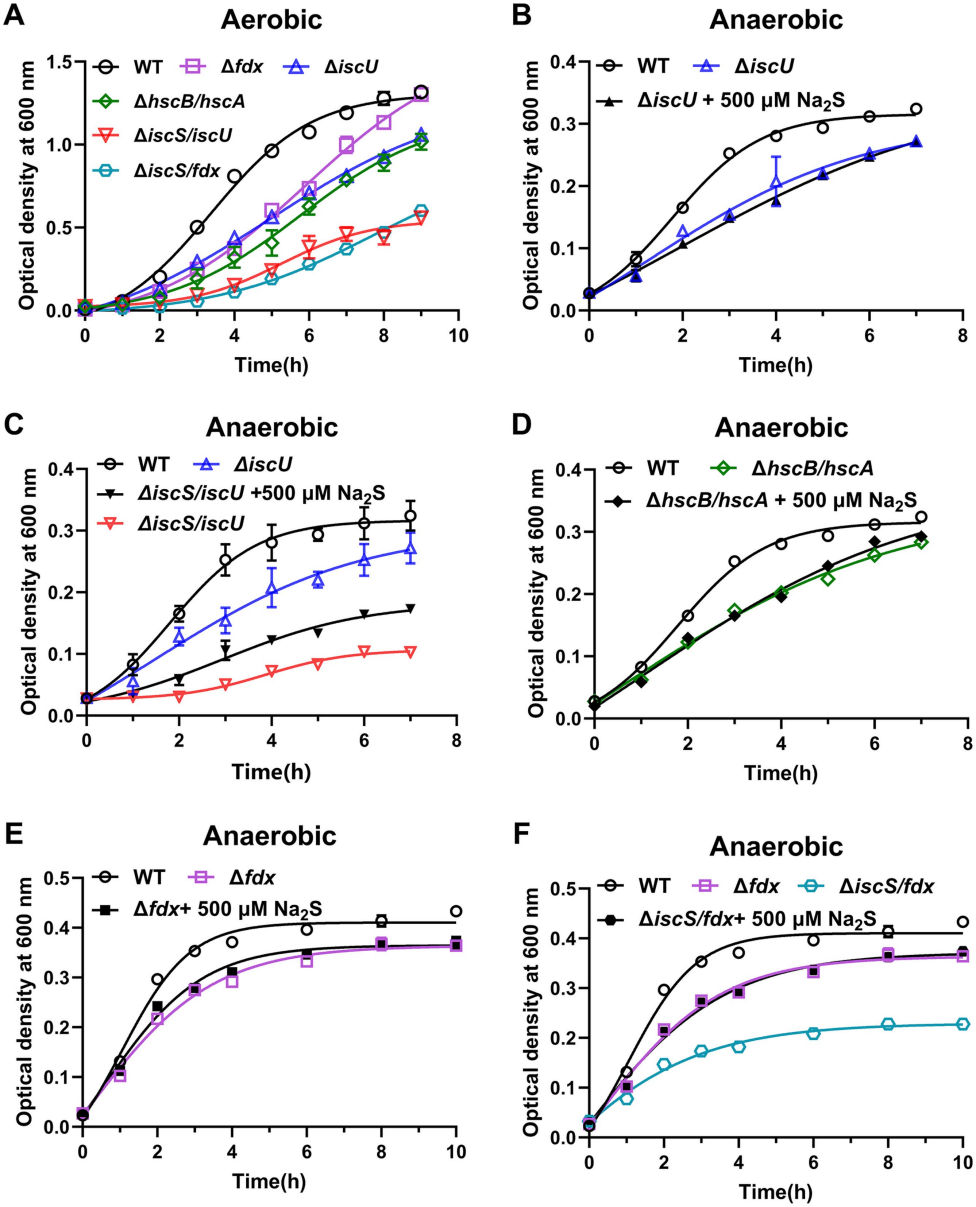
Effects of exogenous H_2_S on the growth of Δ*iscU*, Δ*iscS/iscU*, Δ*hscB/hscA*, Δ*fdx*, and Δ*fdx/iscS* mutant strains. **(A)** Growth curves of the wild-type (WT) *E. coli* strain, Δ*iscU*, Δ*hscB/hscA*, Δ*iscS/iscU*, Δ*fdx*, and Δ*fdx/iscS* mutant strain in LB medium under aerobic conditions, respectively. **(B)** Effects of 500 μM Na_2_S on the growth of the Δ*iscU* mutant strain under anaerobic conditions. **(C)** Effects of 500 μM Na_2_S on the growth of the Δ*iscS/iscU* mutant strain under anaerobic conditions. **(D)** Effects of 500 μM Na_2_S on the growth of the Δ*hscB/hscA* mutant strain under anaerobic conditions. **(E)** Effects of 500 μM Na_2_S on the growth of the Δ*fdx* mutant strain under anaerobic conditions. **(F)** Effects of 500 μM Na_2_S on the growth of the Δ*fdx/iscS* mutant strain under anaerobic conditions. Data are represented as means ± SD (*n* = 6).

Given that the primary role of IscS is to transport sulfur to the scaffold protein IscU for Fe-S cluster assembly. Based on the observations mentioned above, we postulated that H_2_S could serve as a sulfur donor to facilitate Fe-S cluster assembly in the Δ*iscS* mutant cells. Accordingly, we deleted *iscU* in the Δ*iscS* background to generate a Δ*iscS*/*iscU* double mutant. For comparative purposes, we also constructed Δ*iscU*, Δ*fdx*, Δ*hscB*/*hscA*, and Δ*iscS*/*fdx* strains. All genotypes were confirmed by sequencing PCR-amplified fragments from the flanking regions of each target site (Supplementary Figure S4). We assessed the growth of these mutant strains and found that all exhibited markedly impaired growth under both aerobic and anaerobic conditions compared to the wild type (Figure 6).

We next investigated the effect of Na_2_S (500 μM) on the growth rates of these mutant strains under anaerobic conditions. The failure of H₂S to rescue the Δ*iscU* mutant (Figure 6B) demonstrated that the IscU scaffold protein is required for H₂S-mediated Fe-S cluster biosynthesis. This strongly suggests that the sulfur from exogenous H₂S must be delivered to the IscU scaffold to promote Fe-S cluster assembly. While H₂S treatment slightly restored the growth of the Δ*iscS*/*iscU* mutant (Figure 6C). This rescue suggests that H₂S may compensate for additional biological functions of IscS beyond its role in Fe-S cluster assembly, which may include its known activities in tRNA thiolation, thiamine biosynthesis, and molybdopterin synthesis (Mihara and Esaki, 2002). Notably, previous studies have established that sulfide can serve as an alternative sulfur source for both *in vivo* 4-thiouridine synthesis (Lauhon et al., 2004; Gervason et al., 2025b) and *in vitro* molybdopterin synthesis (Leimkühler and Rajagopalan, 2001; Leimkuhler et al., 2003). Given that H₂S-derived sulfane sulfur species (e.g., persulfides/polysulfides) may serve as sulfur donors for both 4-thiouridine and molybdopterin biosynthesis, we sought to determine whether exogenous H₂S treatment could increase cellular sulfane sulfur levels in the Δ*iscS*/*iscU* mutant. To this end, sulfane sulfur was quantified using the fluorescent probe SSP4, which specifically reacts with sulfane sulfur but not with H₂S. Our results demonstrated that H₂S supplementation indeed led to a significant increase in cellular sulfane sulfur levels (Supplementary Figure S5). This increase provides a plausible sulfur source for multiple biosynthetic pathways, including 4-thiouridine and molybdopterin. However, assessment of the Fe-S cluster-dependent enzyme fumarate reductase (FR) activity in the Δ*iscS*/*iscU* mutant showed that it was not significantly affected by 500 μM Na₂S supplementation (Supplementary Figure S6), indicating that neither exogenous H₂S nor the elevated sulfane sulfur pool can rescue Fe-S cluster biogenesis in the absence of the canonical IscS/IscU machinery.

Crucially, H₂S exposure could not rescue the growth of the Δ*hscB*/*hscA* mutant (Figure 6D), which lacks the chaperones required for Fe-S cluster transfer. This rules out the possibility that H₂S directly targets apo-proteins for Fe-S cluster assembly, as occurs during chemical reconstitution *in vitro* (Supplementary Figure S7). In addition, H₂S treatment did not restore growth in the Δ*fdx* mutant (Figure 6E) but rescued the growth rate of the Δ*iscS*/*fdx* double mutant to that of the Δ*fdx* single mutant (Figure 6F), indicating that H₂S can compensate for the functional loss of IscS in the Δ*iscS*/*fdx* mutant. H₂S treatment did not significantly affect the growth of the Δ*iscU*, Δ*fdx*, or Δ*hscB*/*hscA* mutant strains, thereby ruling out the possibility that H₂S acts as a signaling molecule to directly regulate *E. coli* metabolic pathways. This further indicates that the restoration of proliferation in the Δ*iscS* mutant by H₂S occurs primarily through compensating for the Fe-S cluster biosynthesis function of IscS.

### 3.5 Exogenous H₂S increased both the Fe-S cluster content and the enzymatic activity of recombinant aconitase B expressed in the Δ*iscS* mutant

**Figure 7:**
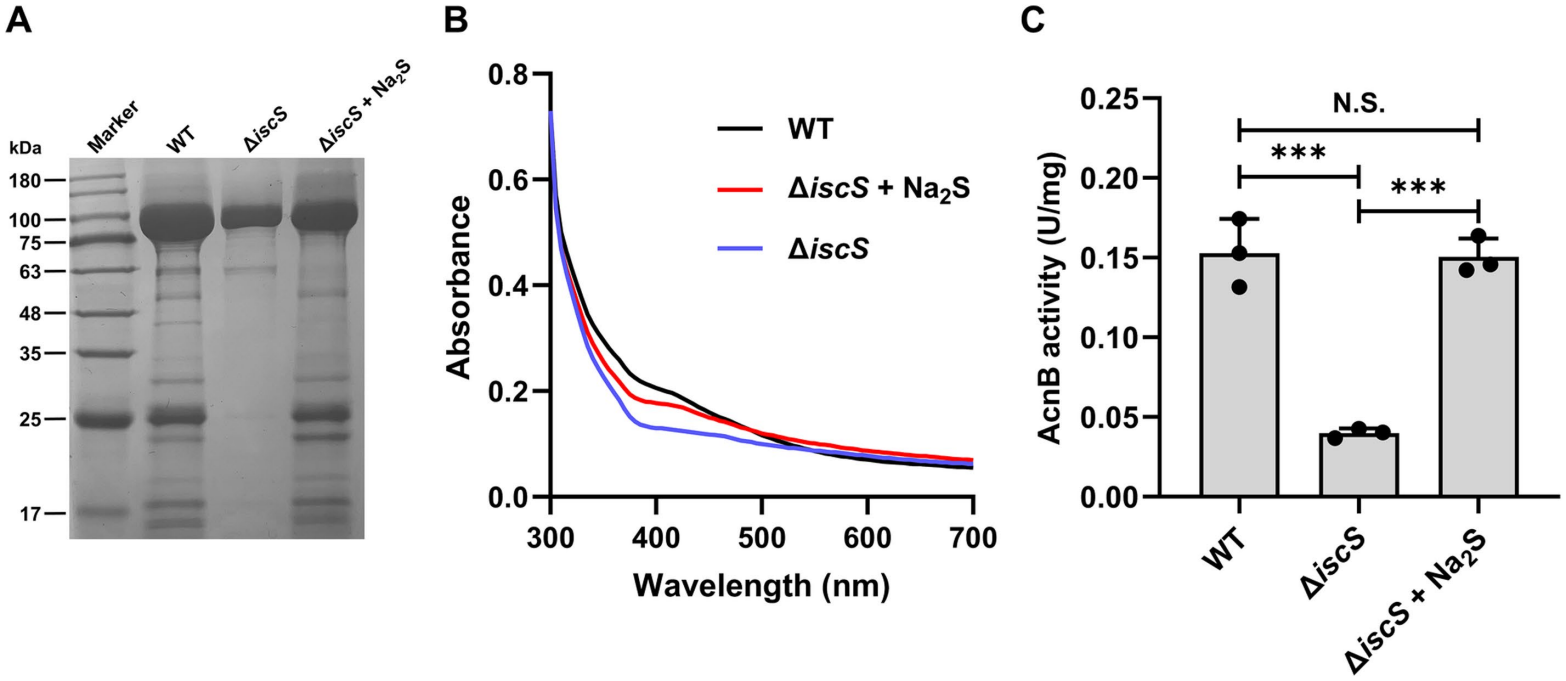
Effects of exogenous H_2_S on the expression and activity of the Fe-S Protein aconitase B (AcnB). **(A)** SDS-PAGE (12%) analysis of the purified AcnB expressed in wild-type *E. coli* (WT) and the Δ*iscS* mutant cells. **(B)** UV–visible absorption spectra of recombinant AcnB (1 mg/mL) purified from *E. coli* (WT) and the Δ*iscS* mutant, respectively. **(C)** The effect of exogenous H_2_S on the activity of AcnB expressed in *E. coli* (WT) and the Δ*iscS* mutant cells. Data are presented as means ± SD (*n* = 3). ***p < 0.001. N.S., not significant.

To further validate that H₂S can act as a sulfur donor for Fe-S cluster biosynthesis, we expressed and purified aconitase B (AcnB) from the Δ*iscS* mutant cultures grown with or without 500 μM Na₂S (Figure 7A). The yield of purified AcnB from the untreated Δ*iscS* mutant was approximately 0.4 mg/L culture, representing a fivefold decrease compared to that obtained from wild-type *E. coli* (~2 mg/L). However, supplementation with Na₂S increased the AcnB yield in the Δ*iscS* mutant to approximately 1 mg/L. As AcnB harbors a [4Fe-4S]^2+^ cluster, the functional enzyme displays a characteristic absorbance at 420 nm (Freibert et al., 2018). The UV–vis spectra of recombinant AcnB purified from the Δ*iscS* mutant showed a significantly reduced absorbance at 420 nm compared to that isolated from the wild-type cells. However, supplementation with Na₂S restored the 420 nm absorbance of AcnB from the Δ*iscS* mutant to a level comparable to that purified from the wild-type cells (Figure 7B). Furthermore, the activity of the purified AcnB from the Δ*iscS* mutant was 0.04 ± 0.003 U/mg, approximately fourfold lower than that from wild-type cells (0.15 ± 0.021 U/mg). As expected, supplementation with Na₂S restored AcnB activity to a level comparable to that purified from the wild-type cells (Figure 7C). Collectively, these results demonstrate that exogenous H_2_S can serve as a sulfur donor to participate in the assembly of Fe-S clusters in the Δ*iscS* mutant under anaerobic conditions.

## 4 Discussion

Fe-S clusters are highly conserved from prokaryotes to eukaryotes and play critical roles in numerous biological processes (Beinert et al., 1997; Zeng et al., 2007). These cofactors can be reconstituted *in vitro* under strict anaerobic conditions using chemical or biochemical methods (Freibert et al., 2018). In such assays, sodium sulfide (Na₂S) is widely employed as an inorganic sulfur source for Fe-S cluster assembly (Kennedy and Beinert, 1988; Freibert et al., 2018). Furthermore, stable Fe-S clusters can self-assemble spontaneously in aqueous solutions containing low concentrations of L-cysteine, FeCl₃, and Na₂S (Jordan et al., 2021). In the present study, we employed aconitase B (an Fe-S cluster-dependent enzyme) as a model system to investigate the *in vitro* chemical reconstitution of Fe-S clusters. Recombinant aconitase B was expressed and purified from *E. coli*, after which its Fe-S prosthetic group was chemically stripped to generate the apoprotein. Using Na₂S as the sole sulfur source, we successfully reconstituted the Fe-S cluster into the apoprotein under anaerobic conditions (Supplementary Figure S7). Notably, the enzymatic activity of the reconstituted aconitase B remained lower than that of the native holoenzyme purified from cells (data not shown), consistent with previous reports (Kennedy and Beinert, 1988).

According to a hypothesis related to the origin of life, the anoxic environment of early Earth, particularly the surroundings of iron- and sulfur-bearing volcanic rocks at alkaline hydrothermal vents, would have favored the spontaneous formation of Fe-S clusters (Jordan et al., 2021). Following the Great Oxidation Event, rising atmospheric oxygen levels diminished the availability of iron and sulfur and introduced oxidative stress that disrupted Fe-S clusters (Jordan et al., 2021). As a result, cells evolved specialized protein systems to protect against oxidative disruption and to efficiently assemble Fe-S clusters under changed biogeochemical conditions (Outten, 2015). Indeed, previous work has established that sulfide rather than cysteine serves as the sulfur source for Fe-S cluster assembly in the methanogenic archaeon (Liu et al., 2010; Dussouchaud et al., 2025). In the present study, although the deletion of *iscS* disrupts the primary machinery for Fe-S cluster biosynthesis, the Δ*iscS* mutant may retain the capacity for spontaneous Fe-S cluster assembly using H₂S as a sulfur source under anaerobic conditions.

Exogenous H₂S restored the growth defect of the Δ*iscS* mutant cultured in both LB medium and M9 minimal medium (Supplementary Figure S8). Expression of the IscS (C328A) mutant protein, in which the catalytic cysteine at position 328 was substituted with alanine, failed to complement the growth defect of the Δ*iscS* mutant, whereas expression of wild-type IscS successfully restored growth (Supplementary Figure S9). In addition, our previous study demonstrated that H₂S promotes ATP synthesis in the Δ*iscS* mutant under anaerobic conditions (Wang et al., 2019), indicating a stimulatory effect on energy metabolism. Here, we conducted integrated proteomic and metabolomic analyses of Δ*iscS* mutant cells treated with or without H₂S. The results showed that the up-regulated Fe-S proteins were predominantly associated with nitrate and fumarate respiration, key pathways supporting energy metabolism under anaerobic conditions (Table 1). Furthermore, given that quinolinate synthase is an Fe-S cluster-dependent enzyme essential for NAD^+^ biosynthesis, we propose that exogenous H₂S enhances NAD^+^ synthesis in the Δ*iscS* mutant, as confirmed by metabolomic data (Figure 4), by promoting the incorporation of Fe-S clusters into this enzyme in the Δ*iscS* mutant. The resulting increase in NAD^+^ pool would facilitate the oxidation of NADH to support ATP synthesis, utilizing NADH as the key electron donor for anaerobic respiration (Hellingwerf et al., 1981; Stewart, 1988). Consistent with this, an elevated succinate level, which is the end product of fumarate respiration, further confirmed that H₂S exposure enhanced energy metabolism in the Δ*iscS* mutant (Figure 4).

The primary function of IscS *in vivo* is to deliver sulfur to assemble Fe-S clusters on the scaffold protein IscU (Baussier et al., 2020; Braymer et al., 2021; Fujishiro et al., 2022). Previous studies have also shown that sodium sulfide (Na₂S) can serve as a sulfur source for Fe-S cluster assembly on IscU *in vitro* (Zeng et al., 2007). We therefore hypothesized that H_2_S could act as a sulfur donor delivered directly to the IscU scaffold to support Fe-S cluster biosynthesis in the Δ*iscS* mutant cells. As expected, H₂S treatment failed to rescue the growth defect of the Δ*iscU* mutant (Figure 6C), indicating that H₂S serves as a sulfur source that depends on delivery through the IscU scaffold for Fe-S cluster assembly. In addition, Na₂S supplementation during recombinant expression of aconitase B in the Δ*iscS* mutant significantly increased its Fe-S cluster abundance and enzymatic activity (Figure 7), further supporting the involvement of H₂S in Fe-S cluster assembly.

In addition to its central role in Fe-S cluster assembly, IscS has other biological functions, including tRNA thiolation, thiamine biosynthesis, and molybdopterin synthesis (Mihara and Esaki, 2002). Although previous studies showed that the growth defect of the Δ*iscS* mutant can be partially rescued by supplementing minimal medium with nicotinic acid, thiamine, and isoleucine (Lauhon and Kambampati, 2000), the addition of these nutrients did not significantly improve the growth of the Δ*iscS* mutant in LB rich medium under anaerobic conditions (Supplementary Figure S10). The slight rescue of the Δ*iscS*/*iscU* double mutant by H₂S treatment may be attributed to the ability of H₂S to compensate for non-Fe-S-cluster-related functions of IscS (Figure 6C), such as its role in tRNA thiolation and molybdopterin synthesis (Mihara and Esaki, 2002). In addition, unlike the elevated mitochondrial iron levels observed in cysteine desulfurase-deficient yeast or mammalian cells (Li et al., 1999; Zhang et al., 2021), no significant difference in cellular iron content was detected between the Δ*iscS* mutant and the wild-type strain (Supplementary Figure S11).

**Figure 8:**
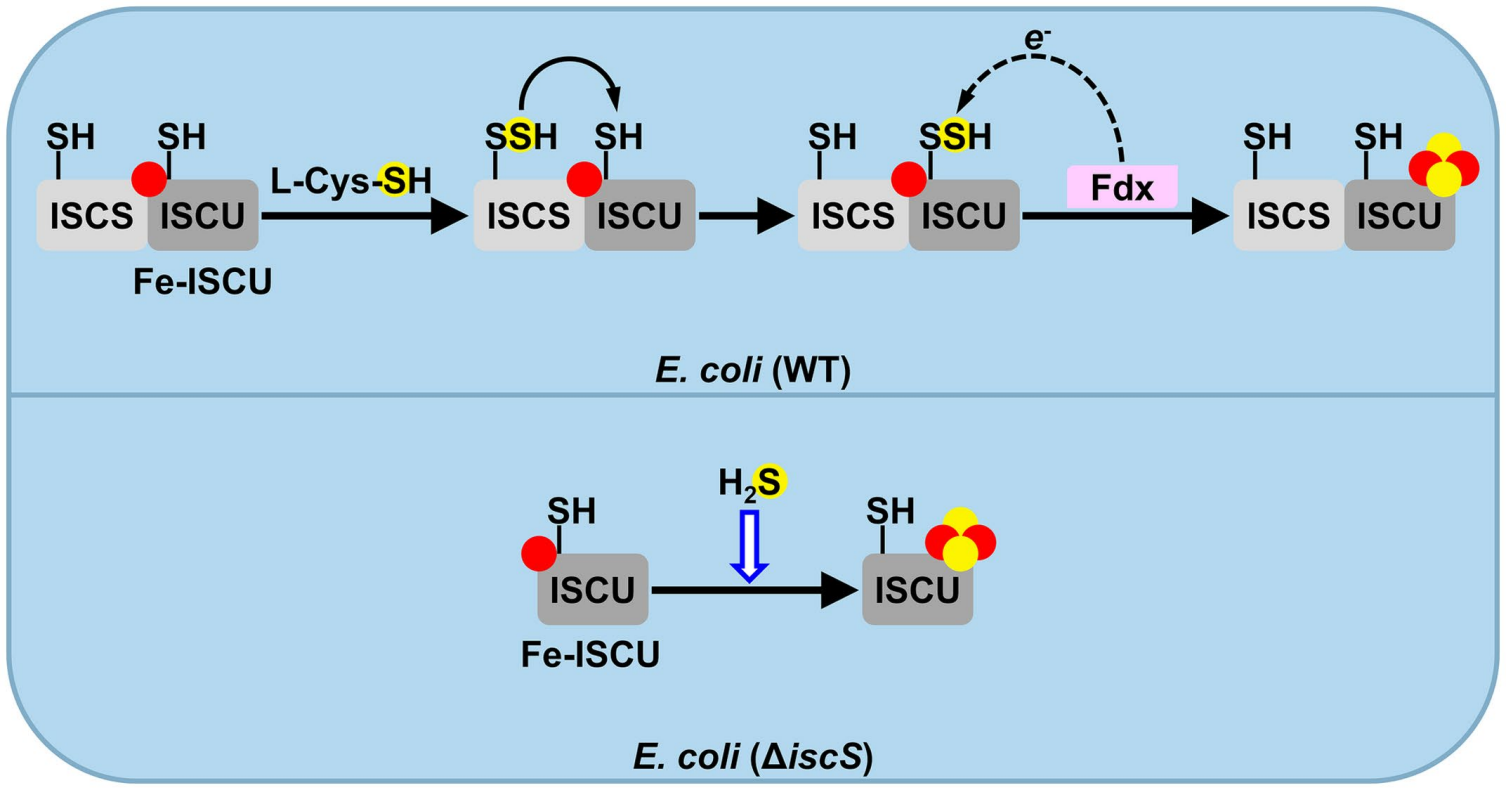
A plausible mechanism for H_2_S serving as a sulfur source to assemble Fe-S clusters in the Δ*iscS* mutant cells. In wild-type (WT) *E. coli* cells, cysteine desulfurase (IscS) catalyzes the conversion of cysteine to form a protein-bound cysteine persulfide intermediate and subsequently delivers sulfur to the scaffold protein IscU. Ferredoxin (Fdx) provides electrons to reduce the persulfide intermediate to sulfide. Alternatively, in the Δ*iscS* mutant strain or those organisms that live in sulfide-rich habitats, H_2_S can serve as a sulfur donor to assemble Fe-S clusters on the iron-containing form of IscU (Fe-IscU).

Since Fe-S clusters can be reconstituted directly onto apoproteins *via* chemical or biochemical methods *in vitro*, we asked whether H₂S could act as a sulfur donor to directly assemble Fe-S clusters on apoproteins within *E. coli* cells. To test this, we constructed a Δ*hscB*/*hscA* double mutant, in which the dedicated chaperone system required for Fe-S cluster transfer is inactivated. The results showed that, unlike chemical reconstitution *in vitro*, H₂S cannot directly target apoproteins for Fe-S cluster assembly *in vivo* (Figure 6D). The assembly of Fe-S clusters on apoproteins *in vitro* requires high concentrations of iron and sulfide under anaerobic conditions. However, the intracellular environment presents two major limitations: first, the concentration of labile iron is very low (~1–3 μM) (Recalcati et al., 2017), and second, the bioavailability of sulfide is restricted. Although the medium is supplemented with 500 μM Na₂S, the sulfide equilibrates rapidly to H₂S and HS^−^. Since only H₂S can diffuse across the membrane, the intracellular pool of sulfide (H₂S/HS^−^) remains low. Therefore, within the complex cytoplasmic environment, the direct assembly of Fe-S clusters on apoproteins is highly challenging. Indeed, a recent study demonstrated that Fe-S cluster assembly is initiated by the insertion of ferrous iron into ISCU to form Fe-bound ISCU, which subsequently triggers the transfer of a persulfide sulfur from NFS1 to Fe-bound ISCU (Srour et al., 2022). Thus, in the Δ*iscS* mutant, intracellular sulfide (H₂S/HS^−^) serves as an alternative sulfur source to facilitate Fe-S cluster assembly, potentially through its action on the Fe-bound ISCU scaffold (Figure 8).

The Fdx protein provides electrons to reduce the persulfide intermediate to sulfide during Fe-S cluster assembly. In the Δ*fdx* mutant, sulfur transfer from IscS to IscU remains functional, which results in the accumulation of the persulfide intermediate on IscU. Consequently, the sulfur-binding site on IscU is occupied, which likely prevents H₂S from directly accessing the site and supplying sulfide for Fe-S cluster assembly. This provides a plausible explanation for why H₂S fails to restore the growth defect in the Δ*fdx* mutant (Figure 6E). In contrast, H₂S treatment restored the growth of the Δ*fdx*/*iscS* double mutant to a level comparable to that of the Δ*fdx* single mutant (Figure 6F), indicating that H₂S serves as a sulfur source participating in Fe-S cluster assembly in the Δ*fdx*/*iscS* mutant. These results indicated that H₂S-mediated restoration of Fe-S cluster biosynthesis does not require Fdx in the Δ*fdx*/*iscS* mutant under anaerobic conditions, which is consistent with prior reports that Fdx is dispensable for Fe-S cluster biosynthesis *via* the ISC system in *E. coli* under anaerobic conditions (Tanaka et al., 2016).

Fumarate and Nitrate reductase Regulatory protein (FNR), an Fe-S cluster-dependent protein, functions as a global transcriptional regulator of genes encoding proteins involved in energy metabolism under anaerobic conditions (Lambden and Guest, 1976; Unden et al., 1994). To determine whether FNR is involved in the H₂S-mediated restoration of the Δ*iscS* mutant growth, we constructed Δ*fnr* and Δ*iscS*/Δ*fnr* mutants (Supplementary Figure S4). Consistent with previous reports (Spiro and Guest, 1990), *fnr* deletion specifically reduced the growth rate only under anaerobic conditions (Supplementary Figure S12). Under anaerobic conditions, the Δ*iscS*/Δ*fnr* double mutant exhibited severe growth defects comparable to those of the Δ*fnr* single mutant, with no significant difference in their growth rates (Supplementary Figure S12). Since *fnr* deletion severely impairs anaerobic growth (Shan et al., 2012), the resulting reduced demand for Fe–S clusters in the Δ*fnr* mutant explains why subsequent *iscS* deletion does not cause a significant additional growth defect. Moreover, exogenous H₂S failed to promote the anaerobic growth of either Δ*fnr* or Δ*iscS*/Δ*fnr* mutants (Supplementary Figure S12). This indicates that the restoration of growth in the Δ*iscS* mutant by H₂S ultimately requires FNR-regulated metabolic pathways. However, the failure of H₂S to rescue the Δ*iscU*, Δ*fdx*, and Δ*hscB*/*hscA* mutants (Figure 6), coupled with its lack of significant effect on wild-type growth, excludes the possibility that H₂S acts by upregulating FNR to enhance energy metabolism, thereby restoring Δ*iscS* growth under anaerobic conditions.

In *E. coli*, Fe-S cluster biosynthesis is primarily carried out by two systems: the ISC and SUF systems (Tanaka et al., 2016; Tanaka et al., 2019). Our results demonstrate the critical role of the ISC system over the SUF system in this process. Specifically, deletion of *sufS*—which encodes the cysteine desulfurase of the SUF system—did not significantly impair bacterial growth under aerobic or anaerobic conditions, nor was growth affected by supplementation with 500 μM Na₂S (Supplementary Figure S13). This predominant role of the ISC system is further supported by the findings of Mettert et al. (2008). Using FNR as a model Fe-S-dependent protein, they showed that under anaerobic conditions, deletion of the ISC system reduced FNR activity by approximately 60%, whereas deletion of the SUF system had no effect. As previously reported, expression of the *suf* operon is upregulated in strains lacking the Isc pathway under anaerobic conditions, suggesting that increased expression of the Suf pathway may be partially responsible for the FNR activity remaining in these strains (Mettert et al., 2008). These findings collectively indicate that the SUF system cannot fully compensate for the Fe-S cluster assembly defect in the Δ*iscS* mutant. Consistent with this limited compensatory role, we found that H₂S exposure did not significantly promote the growth of other ISC-deficient mutants (Δ*iscU* or Δ*hscB*/*hscA*), which retain an intact SUF system. This suggests that the rescue of the Δ*iscS* mutant by H₂S is unlikely to be mediated primarily through upregulation of the *suf* operon, although a contributory function of the SUF system cannot be entirely excluded.

In this study, exogenous H₂S rescued the growth defect of the Δ*iscS* mutant under anaerobic conditions by promoting Fe-S cluster biosynthesis. Notably, human luminal H₂S concentrations range from 0.5 to 3 mM, and it is generated in this anaerobic environment as a byproduct of diverse microbial metabolisms (Wolfson et al., 2022). It remains unknown whether wild-type *E. coli* can also utilize H₂S for Fe-S cluster biosynthesis under such sulfide-rich, anaerobic conditions, and this issue needs to be investigated. In addition, we further investigated whether H₂S could also restore growth under aerobic conditions. The results showed that H₂S only partially restored the proliferation of the Δ*iscS* mutant in the presence of oxygen (Supplementary Figure S14). This limited restoration is likely attributable to the volatility of H₂S and its susceptibility to oxidation under aerobic shaking conditions, and this issue needs to be further investigated.

In summary, our study elucidates the mechanism by which H₂S exposure rescues the proliferation impairment of the Δ*iscS* mutant. Specifically, we demonstrate that exogenous H₂S can serve as a sulfur source to facilitate Fe–S cluster assembly, potentially through its action on the Fe-bound ISCU scaffold in the Δ*iscS* mutant.

## Data Availability

The data presented in this study are publicly available. The data can be found in the The BioStudies database under accession numbers S-BSST1803 and S-BSST1790.

## Footnotes

1
https://www.uniprot.org/

2
https://davidbioinformatics.nih.gov/

3
http://www.genome.jp/kegg/
