# Supplementary material for: Hydrogen sulfide acts as a sulfur source for iron sulfur cluster biosynthesis in cysteine desulfurase-deficient Escherichia coli under anaerobic conditions
Source: Front Microbiol. 2026 Mar 11;17:1759970. doi: 10.3389/fmicb.2026.1759970 (PMC13015791; doi:10.3389/fmicb.2026.1759970)
Supplement: Supplementary file 1 [file Data_Sheet_1.docx]

Supplementary Material


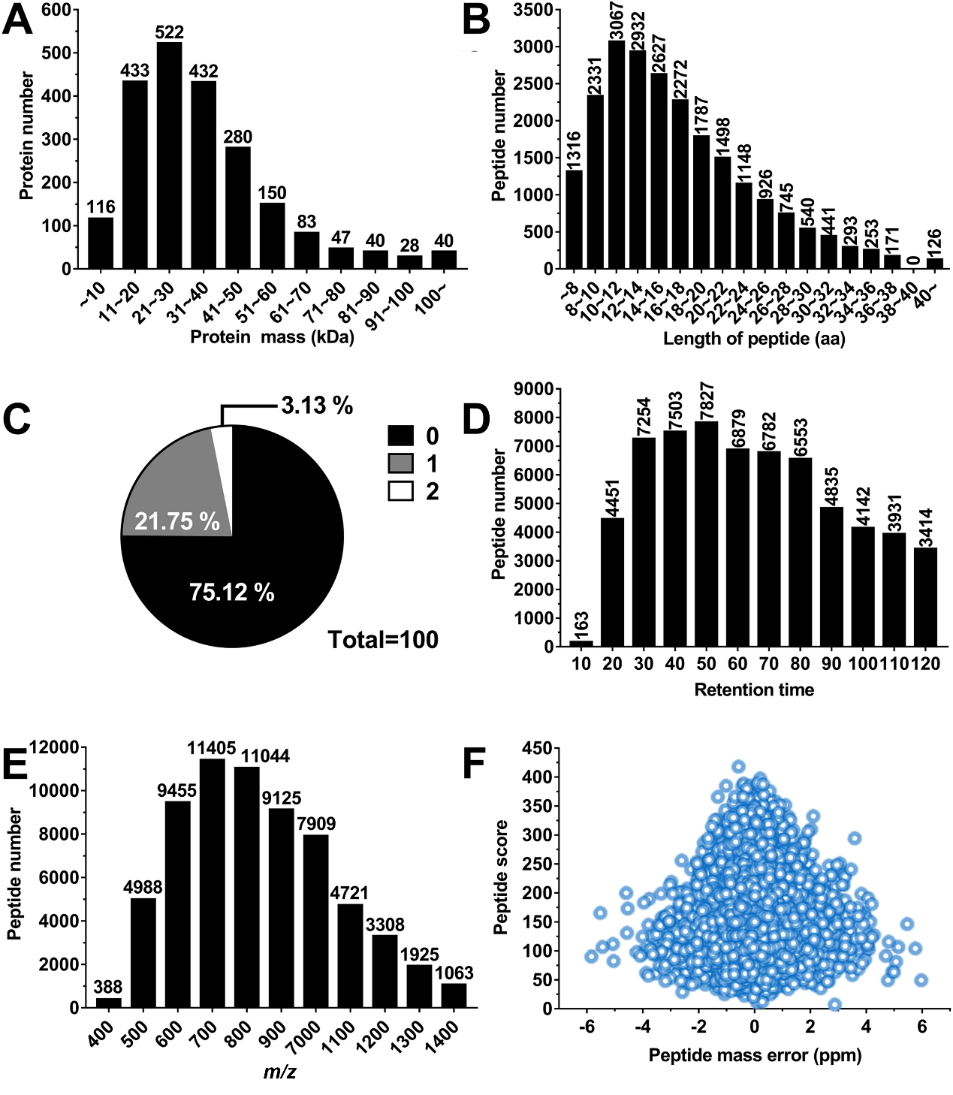


**Figure S1. Reliability analysis of proteomics data.** (A) The molecular weight distribution of the identified proteins. (B) The length distribution of the identified peptides. (C) The percentage of peptides containing different missed cleavage sites. (D) The number of peptides at different retention times by HPLC analysis. (E) The number of peptides at different mass-to-charge ratios (*m/z*). (F) Peptide mass error (ppm) identified by mass spectrometry analysis.


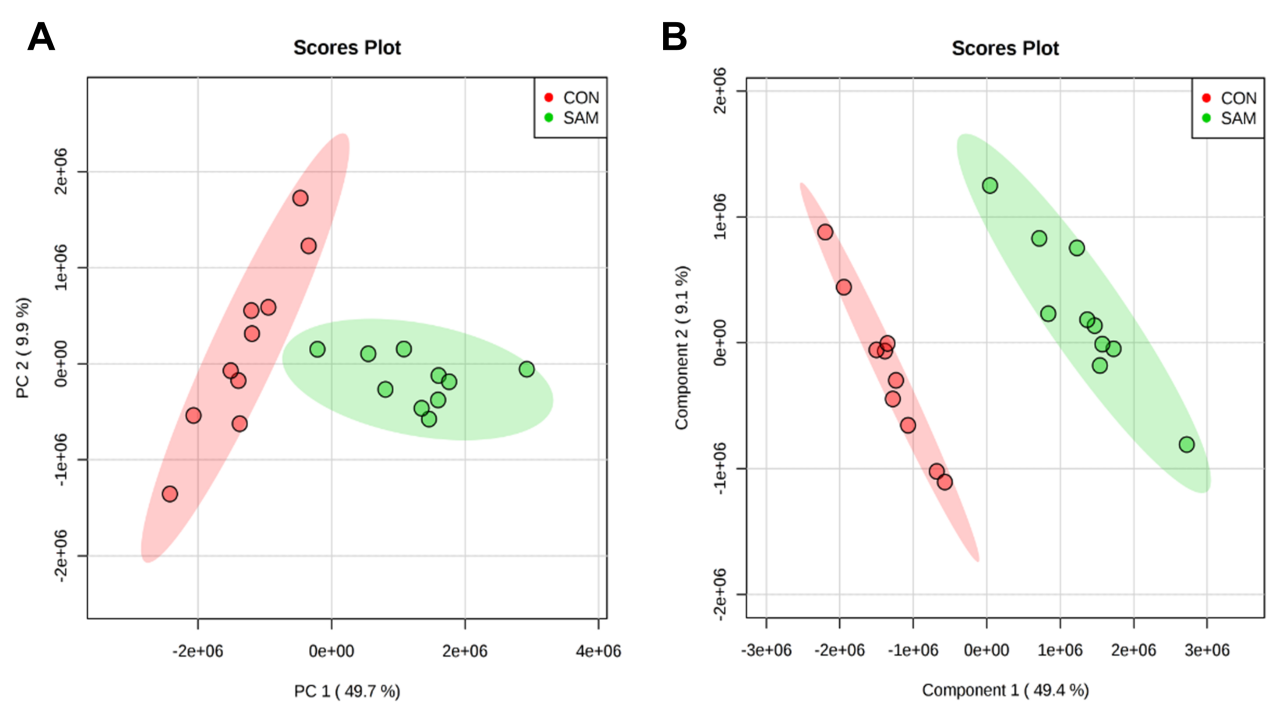


**Figure S2. Reliability analysis of metabolomics.** (A) Principal components analysis (PCA) of metabolites revealed that the two groups with and without H_2_S exposure exhibited distinct aggregation and separation. (B) The partial least squares-discriminant analysis (PLS-DA) demonstrated that the two groups were completely separated. SAM group and CON group represent metabolites of the Δ*iscS* mutant treated with and without 500 μM Na_2_S, respectively.


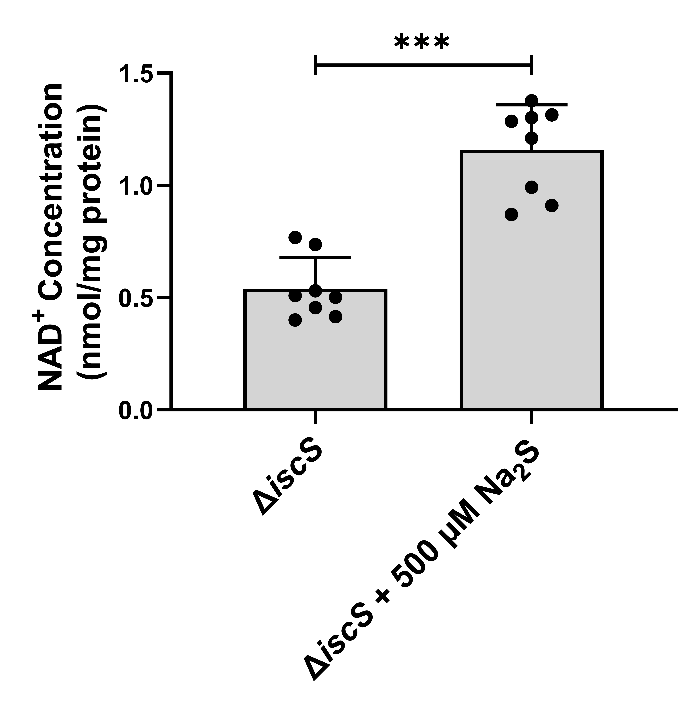


**Figure S3. Concentration of NAD^+^ in the Δ*iscS* mutant treated with or without 500 μM Na_2_S, respectively, under anaerobic conditions.** Data are represented as means ± SD (n = 8). ****p* < 0.001.


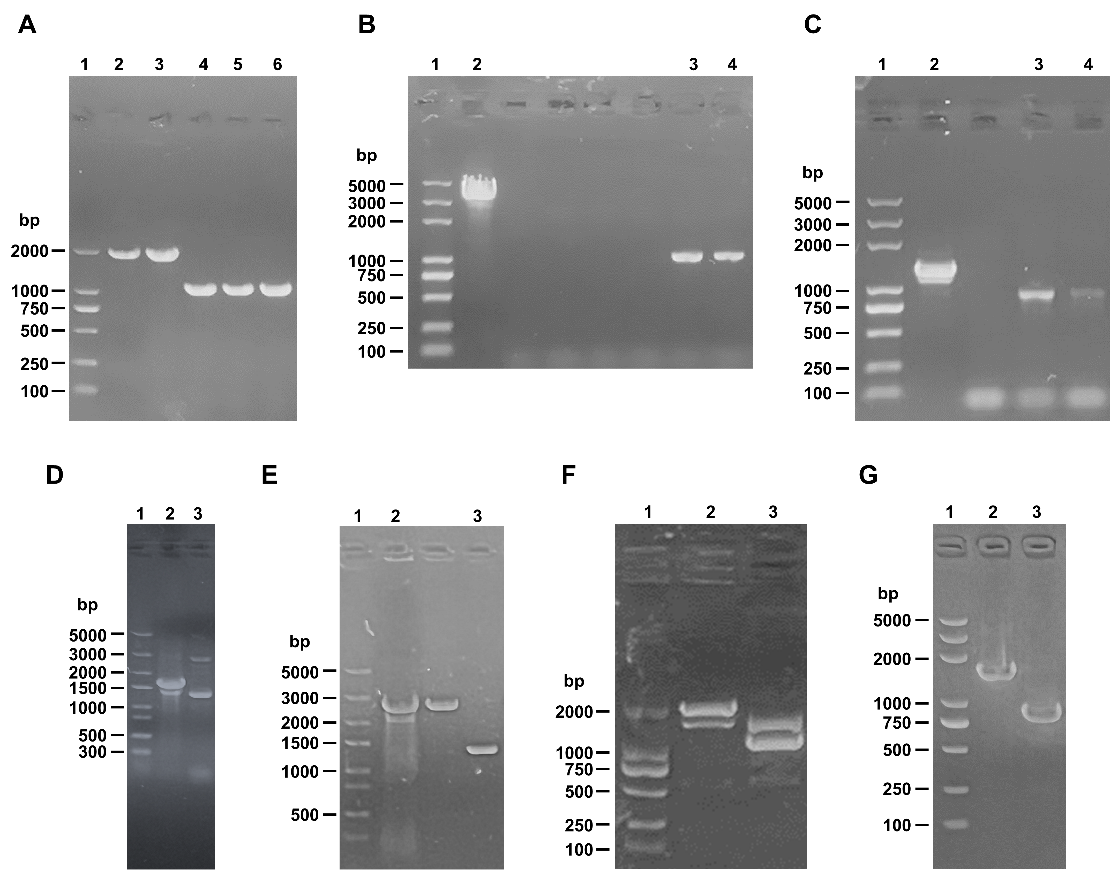


**Figure S4. Verification of the gene deletion mutant strains by PCR.** Validation of the mutant strains was performed by PCR, and then the PCR-amplified fragments from the flanking regions of each target site were separated on an agarose gel for visualization. (A) Validation of the Δ*iscS/iscU* mutant strains by PCR. Lane 1, Molecular mass standards; Lane 2-3, Wild-type *E. coli*; Lane 4-6, the Δ*iscS/iscU* mutant strain. The molecular weight of the PCR products using the wild-type *E. coli* genome as a template should be 1880 bp, and that using the Δ*iscS/iscU* mutant genome as a template should be 1043 bp. (B) Validation of the Δ*hscB/hscA* mutant strains by PCR. Lane 1, Molecular mass standards; Lane 2, Wild-type *E. coli*; Lane 3-4, the Δ*hscA/hscB* mutant strains. The molecular weight of the PCR products using the wild-type *E. coli* genome as a template should be 3401 bp, and that using the Δ*hscA/hscB* mutant genome as a template should be 1018 bp. (C) Validation of the Δ*iscU* mutant strains by PCR. Lane 1, Molecular mass standards; Lane 2, Wild-type *E. coli*; Lane 3-4, the Δ*iscU* mutant strains. The molecular weight of the PCR products using the wild-type *E. coli* genome as a template should be 1315 bp, and that using the Δ*iscU* mutant genome as a template should be 928 bp. (D) Validation of the Δ*fdx* mutant strains by PCR. Lane 1, Molecular mass standards; Lane 2, Wild-type *E. coli*; Lane 3, the Δ*fdx* mutant strain. The molecular weight of the PCR products using the wild-type *E. coli* genome as a template should be 1623 bp, and that using the Δ*fdx* mutant genome as a template should be 1287 bp. (E) Validation of the Δ*fdx/iscS* mutant strains by PCR. Lane 1, Molecular mass standards; Lane 2, the Δ*fdx* mutant strain; Lane 3, the Δ*fdx/iscS* mutant strain. PCR using the Δ*fdx* mutant strain genome as a template produced a 2514 bp fragment, and that using the Δ*fdx/iscS* mutant genome as a template produced a 1299 bp fragment. (F) Validation of the Δ*fnr* mutant strains by PCR. Lane 1, Molecular mass standards; Lane 2, Wild-type *E. coli*; Lane 3, the Δ*fnr* mutant strain. The molecular weight of the PCR products using the wild-type *E. coli* genome as a template should be 1793 bp, and that using the Δ*fnr* mutant genome as a template should be 1040 bp. (G) Validation of the Δ*iscS/fnr* mutant strains by PCR. Lane 1, Molecular mass standards; Lane 2, the Δ*iscS* mutant; Lane 3, the Δ*iscS/fnr* mutant strain. PCR using the Δ*iscS* mutant genome as a template produced a 1667 bp fragment, and that using the Δ*iscS/fnr* mutant genome as a template produced a 914 bp fragment.


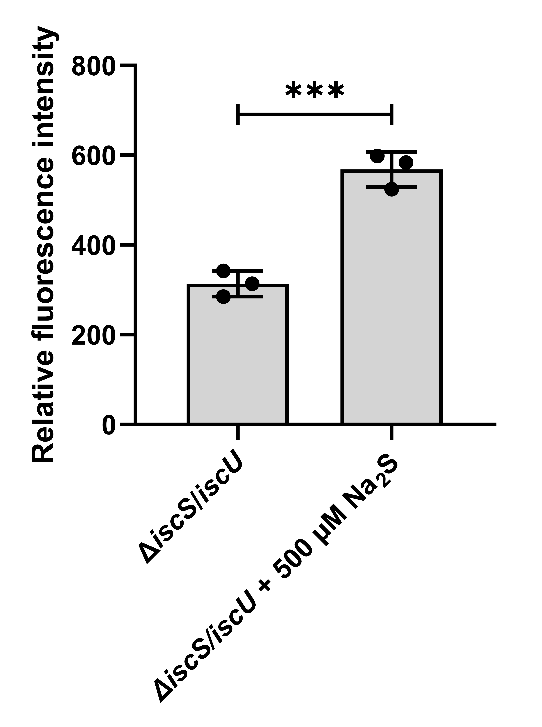


**Figure S5. Effects of exogenous H₂S on the levels of sulfane sulfur species in the Δ*iscS*/*iscU* mutant strains under anaerobic conditions.** Sulfane sulfur species (e.g., persulfides/polysulfides) in the supernatants of lysed *E. coli* cells were quantified using the fluorescent probe SSP4. The procedure was as follows: bacterial cultures in the exponential growth phase were collected by centrifugation at 12,000 rpm for 10 min at 4 ℃ and washed three times with ice-cold 50 mM Tris-HCl buffer (pH 8.0), which was deoxygenated by nitrogen bubbling for 30 min. Next, the cell pellets were resuspended in 50 mM Tris-HCl buffer (pH 8.0) supplemented with lysozyme (0.5 mg/mL) and lysed by six freeze-thaw cycles in liquid nitrogen. The supernatant was obtained by centrifugation at 12,000 rpm for 15 min. The protein concentrations were determined using a BCA assay kit, and all samples were normalized to a final concentration of 5 mg/mL. Aliquots (200 μL) of the normalized samples were then transferred to a 96-well plate, and SSP4 was added to a final concentration of 10 μM. After incubation in the dark at room temperature for 20 min, fluorescence intensities were measured at 515 nm (excitation 482 nm) using a multifunctional microplate reader. The fluorescence values were expressed as the intensity per mg of protein. Data are presented as mean ± SD (n=3). ****p* < 0.001.


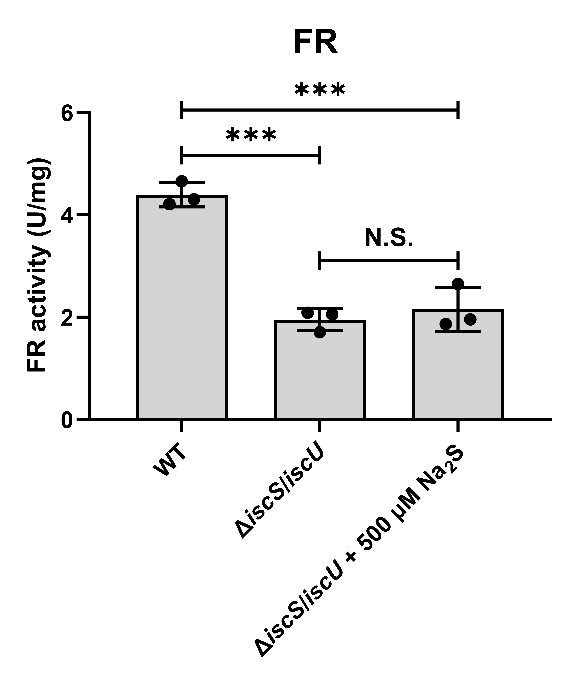


**Figure S6. Effects of exogenous H_2_S on the activity of fumarate reductase (FR) in the Δ*iscS*/*iscU* mutant cells grown in LB medium under anaerobic conditions.** Data are presented as means ± SD (n = 3). ****p* < 0.001; N.S., not significant.


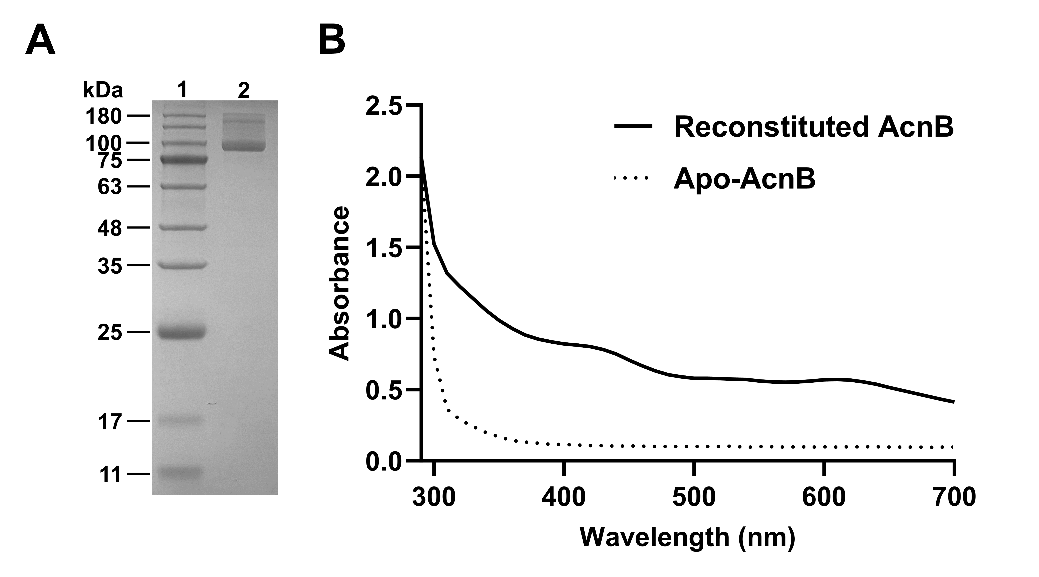


**Figure S7. Chemical reconstitution of Fe-S clusters in purified aconitase B (AcnB) under anaerobic conditions.** (A) SDS-PAGE (12%) analysis of the purified aconitase B (AcnB) protein: Lane 1, Molecular mass standards; Lane 2, Purified Aconitase B (AcnB) protein. (B) UV–visible absorption spectra of the apoprotein Apo-AcnB (2 mg/mL) and the reconstituted AcnB (2 mg/mL).


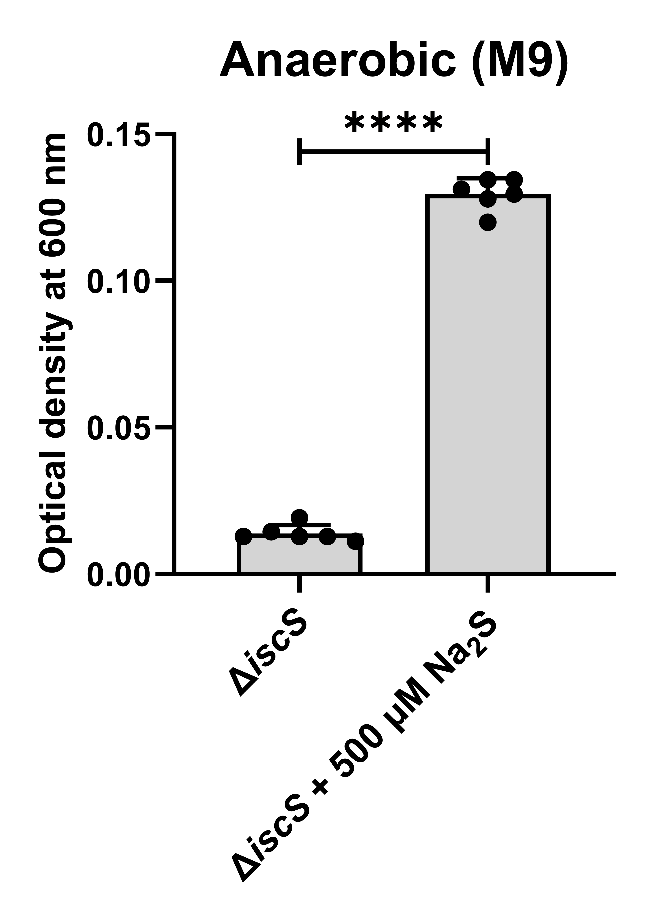


**Figure S8. Effect of 500 μM Na_2_S on the growth of the Δ*iscS* mutant in M9 minimal medium under anaerobic conditions.** The overnight culture was transferred to fresh M9 medium (supplemented with 0.4% (w/v) glucose, thiamine, and trace elements) with 1% (v/v) inoculum. After 12 h of cultivation at 37℃, the optical density at 600 nm (OD_600_) of the culture medium was measured. Blank liquid medium was used as negative control. Data are presented as means ± SD (n = 6). *****p* < 0.0001.


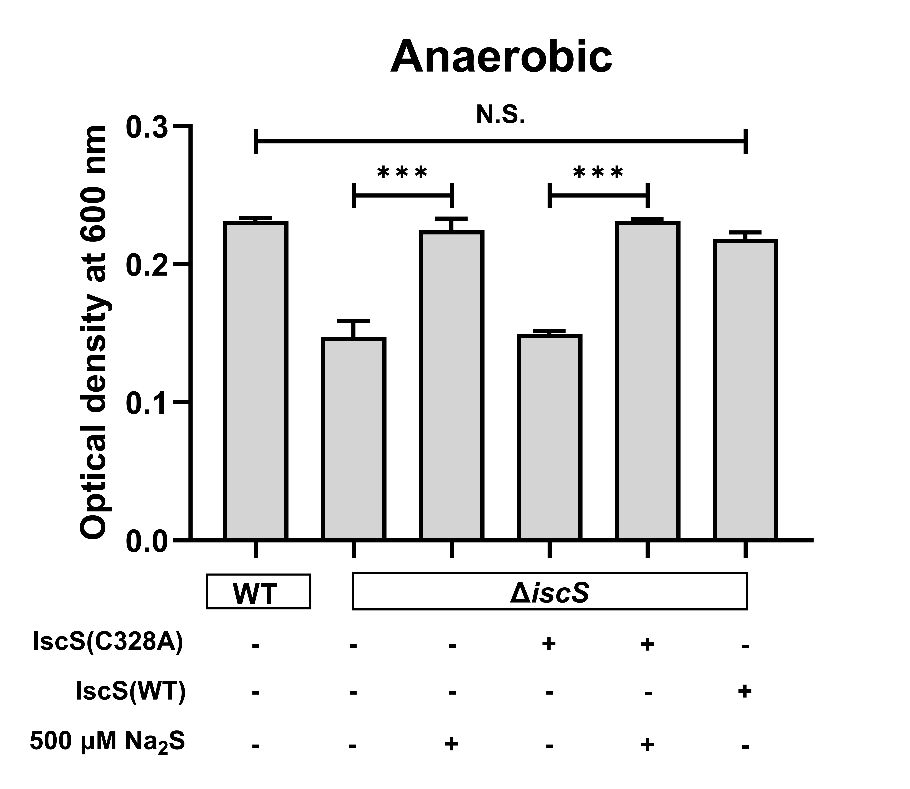


**Figure S9. Expression of wild type IscS (WT) and the IscS (C328A) Mutant in *E. coli* BW25113 (Δ*iscS*).** Effects of complementation with IscS(WT), IscS(C328A) on the growth of the Δ*iscS* mutant in LB medium under anaerobic conditions. After 9 h of cultivation, the optical density at 600 nm (OD_600_) of the culture medium was measured. Blank liquid medium was used as negative control. Data are presented as means ± SD (n = 6). ****p* < 0.001. N.S., not significant.


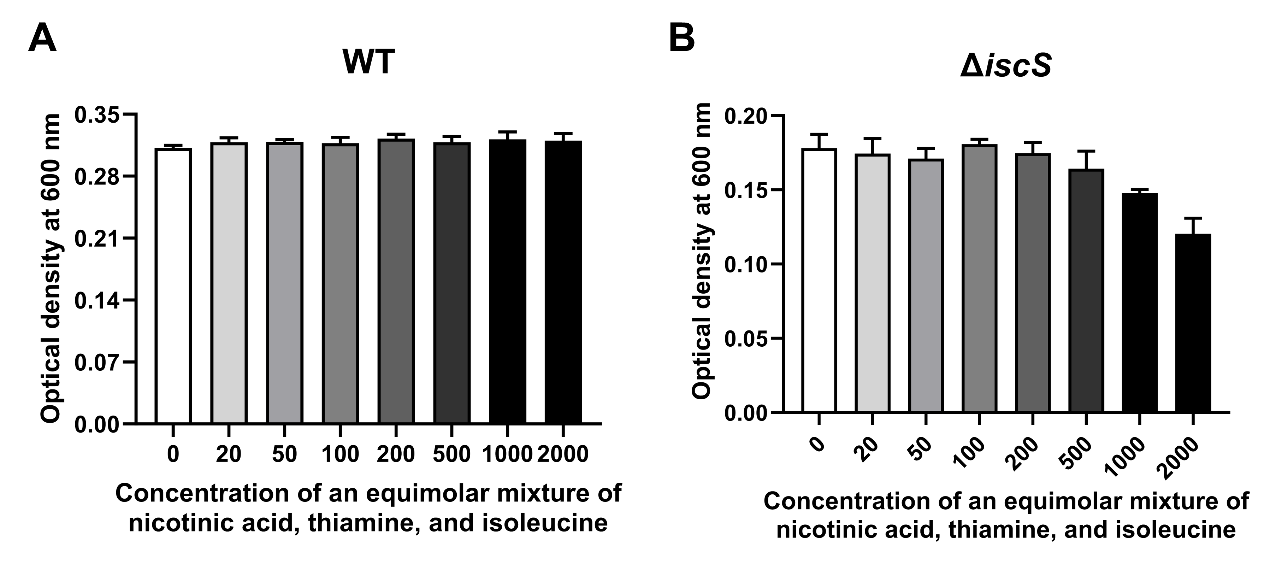


**Figure S10.** **Effects of varying concentrations of nutrients (equimolar mixture of nicotinic acid, thiamine, and isoleucine) on the growth of the wild-type (WT) and the Δ*iscS* mutant strains.** (A) Wild-type (WT) *E. coli* strain. (B) the Δ*iscS* mutant strain. The overnight culture was transferred to fresh LB medium (supplemented with an equimolar mixture of nicotinic acid, thiamine, and isoleucine) with 1% (v/v) inoculum. After 4 h of cultivation in LB medium under anaerobic conditions at 37℃, the optical density at 600 nm (OD_600_) of the culture medium was measured. Blank liquid medium was used as negative control. Data are presented as means ± SD (n = 6).


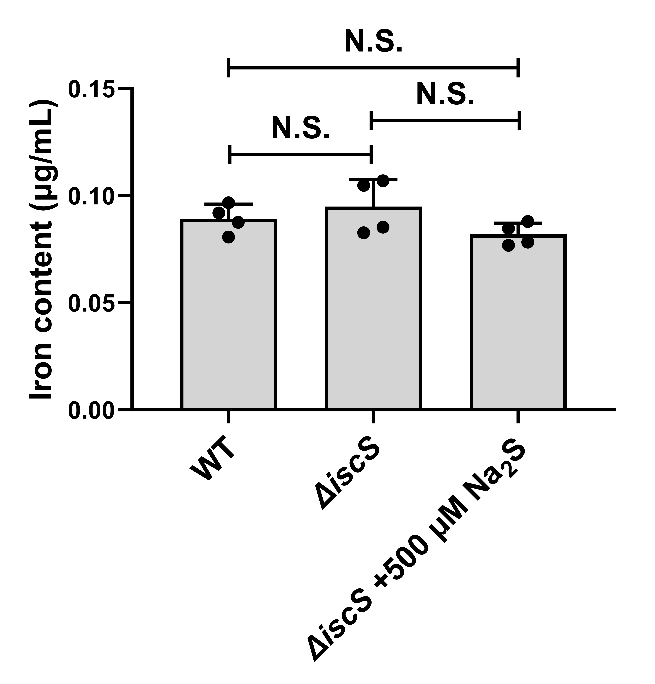


**Figure S11. Determination of iron content in wild type (WT) and the Δ*iscS* mutant strain treated with or without 500 μM Na_2_S.** After being cultured anaerobically in LB medium to exponential phase (OD_600_ ~ 0.2), *E. coli* culture was normalized to an OD_600_ of 0.15. Intracellular iron content was then quantified by atomic absorption spectroscopy. Data are presented as mean ± SD (n=4). N.S., not significant.


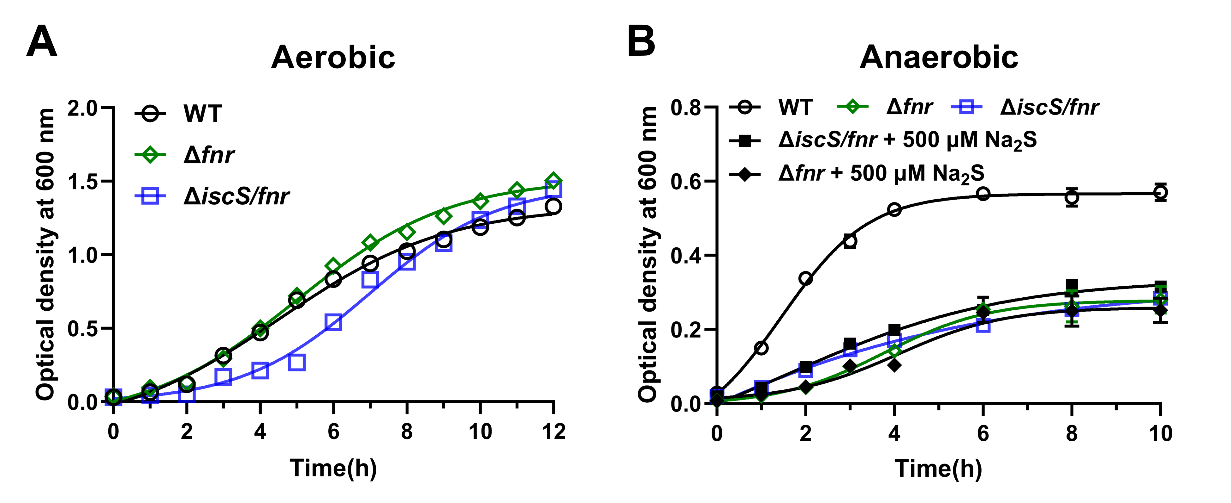


**Figure S12. Effects of exogenous H_2_S on the growth of Δ*fnr* and Δ*iscS/fnr* mutant strains.** (A) Growth curves of the wild-type (WT) *E. coli* strain, Δ*fnr*, and Δ*iscS/fnr* mutant strains under aerobic conditions. (B) Effects of 500 μM Na_2_S on the growth of Δ*fnr* and Δ*iscS/fnr* mutant strains under anaerobic conditions. Data are represented as means ± SD (n = 6).


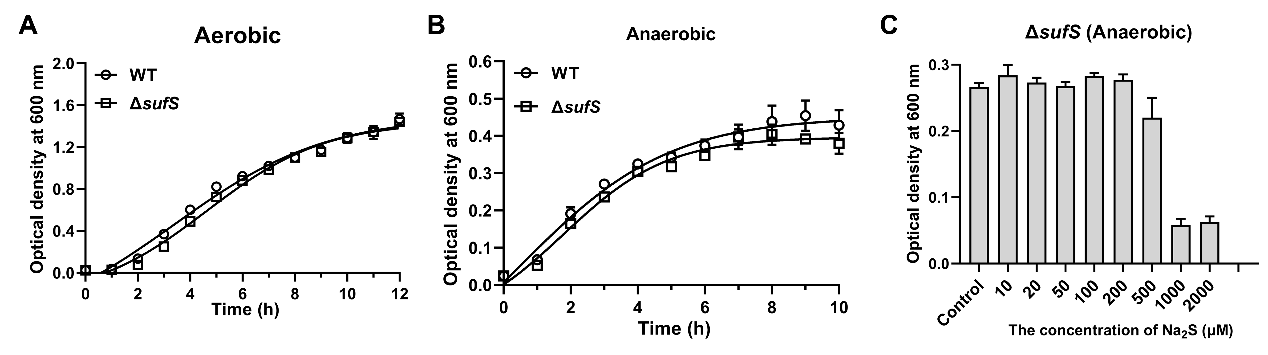


**Figure S13. Deletion of the *sufS* gene encoding a cysteine desulfurase in the SUF system did not significantly affect the growth rate of *E. coli* under both aerobic and anaerobic conditions.** (A-B) Growth curves of the wild-type (WT) and the Δ*sufS* mutant strains under aerobic or anaerobic conditions, respectively. (C) Effects of different concentrations of Na_2_S on the growth of the Δ*sufS* under anaerobic conditions. The overnight culture was transferred to fresh LB medium with 1% (v/v) inoculum. After 4 h of cultivation under anaerobic conditions at 37℃, the optical density at 600 nm (OD_600_) of the culture medium was measured. Blank liquid medium was used as negative control. Data are represented as means ± SD (n = 6).


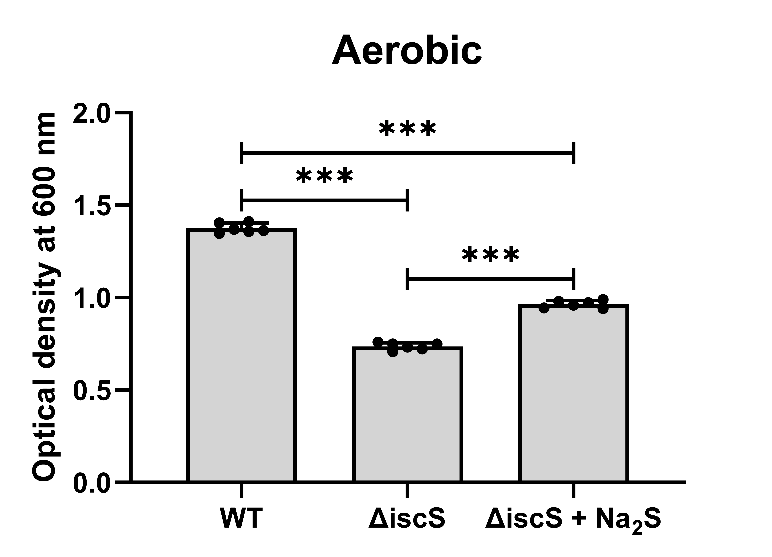


**Figure S14. Effects of exogenous H_2_S on the growth of the Δ*iscS* mutant strain under aerobic conditions.** The overnight culture of the Δ*iscS* mutant strain was transferred to fresh LB medium with 1% (v/v) inoculum. The culture was grown at 37℃ with shaking at 180 rpm. From the time of inoculation, 100 μM Na₂S was added to the medium every hour. After 6 h of cultivation, the optical density at 600 nm (OD_600_) was measured. Blank liquid medium was used as negative control. Data are represented as means ± SD (n = 6). ****p* < 0.001.

**Table S1. Strains and vectors used in this study**

| **Strains** | **Description** |
| --- | --- |
| *E. coli* BW25113 | Preserved in our laboratory |
| *E. coli* BW25113 (Δ*iscS*) | Preserved in our laboratory (1) |
| *E. coli* BW25113 (Δ*iscU*) | This study builds |
| *E. coli* BW25113 (Δ*sufS*) | Preserved in our laboratory (1) |
| *E. coli* BW25113 (Δ*hscA/hscB*) | This study builds |
| *E. coli* BW25113 (Δ*iscS/iscU*) | This study builds |
| *E. coli* BW25113 (Δ*fdx*) | This study builds |
| *E. coli* BW25113 (Δ*fdx/iscS*) | This study builds |
| *E. coli* BW25113 (Δ*fnr*) | This study builds |
| *E. coli* BW25113 (Δ*iscS/fnr*) | This study builds |
| *E. coli* DH5α | Used for the construction of an expression vector |
| *E. coli* BL21 (DE3) | Used for the overexpression of aconitase B |
| **Plasmids** | **Description** |
| pEcCas | Used for the construction of mutant strains |
| pEcgRNA | Used for the construction of mutant strains |
| pET28a | The recombinant expression vector in *E. coli* |
| pSE380 | The recombinant expression vector in *E. coli* |
| pBAD43 | Used for IscS and IscS(C328A) complementation to the Δ*iscS* mutant |

1. Wang J, Guo X, Li H, Qi H, Qian J, Yan S, Shi J, Niu W. 2019. Hydrogen sulfide from cysteine desulfurase, not 3-mercaptopyruvate sulfurtransferase, contributes to sustaining cell growth and bioenergetics in *E. coli* Under Anaerobic Conditions. Front Microbiol **10**:2357.

**Table S2. Primers used in this study**

| **Name** | **Sequence** | **Description** |
| --- | --- | --- |
| **Primers for the construction of *E. coli* mutant strains** | | |
| iscS/iscU N20-F | TAATACTAGTGAGGCTTGCTGACGTACAGGGTTTTAGAGCTAGAAATAGC | Used for the construction of the Δ*iscS/iscU* mutant strain |
| iscS/iscU N20-R | GCTCTAAAACCCTGTACGTCAGCAAGCCTCACTAGTATTATACCTAGGAC |  |
| iscS/iscU-HA-up-F | AGCAAAACCGAACACAAAG |  |
| iscS/iscU-HA-up-R | GCTGGAAGCGATAGCGGACAGGTCAACTTTCAACTG |  |
| iscS/iscU-HA-down-F | CAGTTGAAAGTTGACCTGTCCGCTATCGCTTCCAGC |  |
| iscS/iscU-HA-down-R | ACGCCCAGACGCAGGCCA |  |
| V-iscS/iscU-F | CGGTAGCCTGATTCCTTG |  |
| V-iscS/iscU-R | CGACTTTCACGCCTTTGT |  |

**Table S2 (Continued)**

| iscU N20-F | TAATACTAGTTTACGAGAATCCGCGTAACGGTTTTAGAGCTAGAAATAGC | Used for the construction of the *E. coli* (Δ*iscU*) mutant strain |
| --- | --- | --- |
| iscU N20-R | GCTCTAAAACCGTTACGCGGATTCTCGTAAACTAGTATTATACCTAGGAC |  |
| iscU-HA-up-F | ATGGCGCTGAAAGACCTC |  |
| iscU-HA-up-R | ATAACCAAACCTCAACTCTATAAATTCTCCTGATTC |  |
| iscU-HA-down-F | GAATCAGGAGAATTTATAGAGTTGAGGTTTGGTTAT |  |
| iscU-HA-down-R | ACGTTCGGGTTGGTGAAT |  |
| V-iscU-F | GCGCTGTATGTACGTCGTAAACCGC |  |
| V-iscU-R | GCTTTCGCCGCAACCACA |  |
| hscA/hscB N20-F | TAATACTAGTGCGTCATCCGTTAATGCGCGGTTTTAGAGCTAGAAATAGC | Used for the construction of the  *E. coli* (Δ*hscA/hscB*) mutant strain |
| hscA/hscB N20-R | GCTCTAAAACCGCGCATTAACGGATGACGCACTAGTATTATACCTAGGAC |  |

**Table S2 (Continued)**

| hscA/hscB-HA-up-F | TGTACTGGAATTTGTTGAC | Used for the construction of the *E. coli* (Δ*hscA/hscB*) mutant strain |
| --- | --- | --- |
| hscA/hscB-HA-up-R | ATAACAATCTTTGGCATAAACATTCTCAGGGCTGCG |  |
| hscA/hscB-down-F | CGCAGCCCTGAGAATGTTTATGCCAAAGATTGTTAT |  |
| hscA/hscB-down-R | ATTTCGACTACTAAATCTTC |  |
| V-hscA/hscB-F | GAATGGGTGAAAGGGAAGTCTCTCG |  |
| V-hscA/hscB-R | GATCAAGATCGGGATACGC |  |
| fnr N20-F | TAATACTAGTCGTCAGGCGGAATTCACGAGGTTTTAGAGCTAGAAATAGC | Used for the construction of  *E. coli* (Δ*fnr*) mutant strain |
| fnr N20-R | GCTCTAAAACCTCGTGAATTCCGCCTGACGACTAGTATTATACCTAGGAC |  |
| fnr-HA-up-F | ACCTTGCCATCGGGTTATTG |  |
| fnr-HA-up-R | GTGAGTTATGCGGAAAAAAGGTCTGCTCAAGCCGTAATTG |  |

**Table S2 (Continued)**

| fnr-HA-down-F | CAATTACGGCTTGAGCAGACCT TTTTTCCGCATAACTCAC | Used for the construction of the  Δ*fnr* mutant strain |
| --- | --- | --- |
| fnr-HA-down-F | GTGGTCATTTCGTATGAG |  |
| V-fnr-F | ATTGATACGCTTCCCACT |  |
| V-fnr-R | CGTAACAGATGCCAGTCC |  |
| iscS/fnr N20-F | TAATACTAGTATTATACGGCGCATTCAGTCGTTTTAGAGCTAGAAATAGC | Used for the deletion of the *fnr* gene in the Δ*iscS* mutant strain |
| iscS/fnr N20-R | GCTCTAAAACGACTGAATGCGCCGTATAATACTAGTATTATACCTAGGAC |  |
| iscS/fnr-HA-up-F | TACGCTTCCCACTGCTACGG |  |
| iscS/fnr-HA-up-R | GTTATGCGGAAAAAAGGTCTGCTCAAGCCGTAAT |  |
| iscS/fnr-HA-down-F | CGGCTTGAGCAGACCTTTTTTCCGCATAACTCACTAT | Used for the deletion of the *fnr* gene in the Δ*iscS* mutant strain |
| iscS/fnr-HA-down-F | GACGGTTATGCCAGACCACT |  |
| V-iscS/fnr-F | TACGCTTCCCACTGCTACGG |  |

**Table S2 (Continued)**

| V-iscS/fnr-R | GACGGTTATGCCAGACCACT |  |
| --- | --- | --- |
| fdx N20-F | TAATACTAGTGCATGGTTGATAGTGTAACGGTTTTAGAGCTAGAAATAGC | Used for the construction of  *E. coli* (Δ*fdx*) mutant strain |
| fdx N20-R | GCTCTAAAACCGTTACACTATCAACCATGCACTAGTATTATACCTAGGAC |  |
| fdx-HA-up-F | GCTATGCCGAGCAGGACG |  |
| fdx-HA-up-R | GTCCCATACTAACCTCTGATTAAACCTCGTCCACGG |  |
| fdx-HA-down-F | CCGTGGACGAGGTTTAAT CAGAGGTTAGTATGGGAC |  |
| fdx-HA-down-R | GAGTGAAGCCCCAGGAGC |  |
| V-fdx-F | GATGGTGGGCGGTTCTAC |  |
| V-fdx-R | TCGGCAGGTTGGGTAGAG |  |
| iscS N20-F | TAATACTAGTGAGGTAAAGACGATTTCACGGTTTTAGAGCTAGAAATAGC | Used for the deletion of the *iscS* gene in the *E. coli* (Δ*fdx*) mutant strain |
| iscS N20-R | GCTCTAAAACCGTGAAATCGTCTTTACCTC ACTAGTATTATACCTAGGAC |  |
| iscS-HA-up-F | GGCGATAAATGCCTGACC |  |
| iscS-HA-up-R | CTCCTGATTCCGATACCGATGCTCTATAAACTCCGTAC | Used for the deletion of the *iscS* gene in the *E. coli* (Δ*fdx*) mutant strain |
| iscS-HA-down-F | GTACGGAGTTTATAGAGCA TCGGTATCGGAATCAGGAG |  |
| iscS-HA-down-R | GGTGTTTTTGATCGCCTG |  |
| V-iscS-F | TCAGACTTGACCCTGCTATG |  |
| V-iscS-R | CGACTTTCACGCCTTTGT |  |
| IscS-F | ATGAAATTACCGATTTATCTC | Construction of pBAD43‑*iscS* and pBAD43‑*iscS*(C328A) Plasmids |
| IscS-R | ATGATGAGCCCATTCGATG |  |
| F-328 | AGGTTCCGCCGCGACGTCAGCAAGCCTCGAACC |  |
| R-328 | TTGCTGACGTCGCGGCGGAACCTGAAGAAACTG |  |
| **Primers for qPCR** | | |
| 16S-F | GACGCTCAGGTGCGAAAG | *16S* |
| 16S-R | CCGCTGGCAACAAAGGAT |  |
| frdB-F | GGTATGAAGGTTGAAGCGTTAG | *frdB* |
| frdB-R | CGGACCGATGAACTCTGG | *frdB* |
| narH-R | GCCGCCTCTGTCTCCGATTC | *narH* |
| narH-F | TCTCCTGTGCCTGGGCTTCG |  |
| sdhB-F | TGGAAGCGGATGAAGGTC | *sdhB* |
| sdhB-R | CAGAAAGACGGACAAGAGGT |  |
| lipA-F | TATCCAGGGCATCAAAGC | *lipA* |
| lipA-R | TCAGAATATCCAGAGCACGA |  |
| pykA-F | GACATCATCCTCGCCTCT | *pykA* |
| pykA-R | ACGCCTTTCAGGTGGTTA |  |
| ldhA-F | GATGATGACGCTGAACCG | *ldhA* |
| ldhA-R | TCCATACCCAACGAACCA |  |
| ppc-F | GCTGGAACTGGTCCTCACG | *ppc* |
| ppc-R | GCCCATCCACGAAGTAAAA |  |
| fumA-F | CCAACGGAAGGGAATGAG | *fumA* |
| fumA-R | GCGACAACTGTGCGAGGA |  |
| maeB-F | CGGTGATGGAAGGCAAGG | *maeB* |
| maeB-R | CGCAAACCACGATGTTATGTT |  |
| mdH-F | GGTGAAGATGCGACTCCG | *mdH* |
| mdH-R | CCTGGCTGTTTGCCTTTC | *mdH* |
